# Endo-lysosomal dysregulations and late-onset Alzheimer’s disease: impact of genetic risk factors

**DOI:** 10.1186/s13024-019-0323-7

**Published:** 2019-06-03

**Authors:** Zoë P. Van Acker, Marine Bretou, Wim Annaert

**Affiliations:** 1 Laboratory for Membrane Trafficking, VIB Center for Brain & Disease Research, 3000 Leuven, Belgium; 2 Department of Neurosciences, KU Leuven, Gasthuisberg, O&N4, Rm. 7.159, Herestraat 49, B-3000 Leuven, Belgium

**Keywords:** Autophagy, Endolysosomal flux, LOAD risk factors, Alzheimer’s disease

## Abstract

Increasing evidence supports that cellular dysregulations in the degradative routes contribute to the initiation and progression of neurodegenerative diseases, including Alzheimer’s disease. Autophagy and endolysosomal homeostasis need to be maintained throughout life as they are major cellular mechanisms involved in both the production of toxic amyloid peptides and the clearance of misfolded or aggregated proteins. As such, alterations in endolysosomal and autophagic flux, as a measure of degradation activity in these routes or compartments, may directly impact as well on disease-related mechanisms such as amyloid-β clearance through the blood-brain-barrier and the interneuronal spreading of amyloid-β and/or Tau seeds, affecting synaptic function, plasticity and metabolism. The emerging of several genetic risk factors for late-onset Alzheimer’s disease that are functionally related to endocytic transport regulation, including cholesterol metabolism and clearance, supports the notion that in particular the autophagy/lysosomal flux might become more vulnerable during ageing thereby contributing to disease onset. In this review we discuss our current knowledge of the risk genes APOE4, BIN1, CD2AP, PICALM, PLD3 and TREM2 and their impact on endolysosomal (dys)regulations in the light of late-onset Alzheimer’s disease pathology.

**Electronic supplementary material:**

The online version of this article (10.1186/s13024-019-0323-7) contains supplementary material, which is available to authorized users.

## Background

Alzheimer’s disease (AD) is a progressive neurodegenerative disorder. Patients present with memory dysfunctions and increasing cognitive impairments as disease progresses. There still exists an unmet medical need for effective treatment options, making the prognosis on the patient’s quality of life to be poor after diagnosis. The success rate of AD-clinical trials performed up to 2014 only attained ~ 1%, with even a smaller amount of treatments being effectively FDA-approved [1]. Most of our knowledge of the AD pathology has been obtained from patients with early-onset AD (EOAD, < 65 years), though this group merely makes up for a few percent of the AD cases. AD within this group is generally caused by highly penetrant mutations in a limited set of genes, *PSEN1*, *PSEN2*, and *APP*; with heritability ranging between 92 and 100% [2]. On the contrary, late-onset AD (LOAD), in which patients develop clinical signs after the age of 65, is generally not linked to one specific neuropathological disease cascade or gene mutation. LOAD is likely caused by the co-occurrence of multiple low penetrance risk factor variants in one’s genome in combination with environmental factors; only becoming harmful due to the build-up of cellular stress that is caused by cellular dysfunctions or non-optimal mechanisms linked to these variants. As such, LOAD risk factors refer to those genes (and gene products) of which genetic variation within the population causes some individuals to have a higher predisposition towards the development of LOAD as others.

**Fig. 1:**
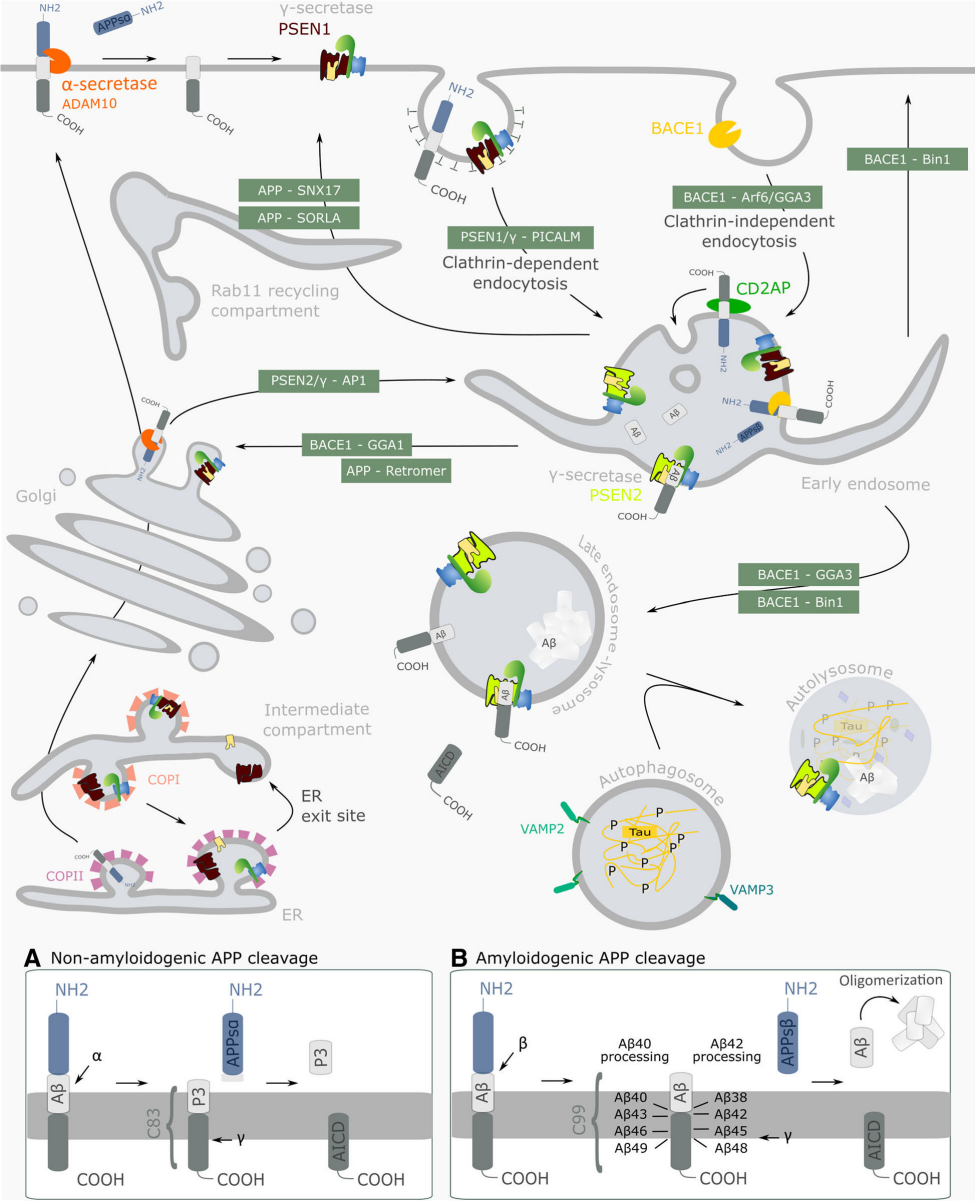
Spatial regulation of non-amyloidogenic and amyloidogenic processing of APP. Dysregulated amyloid precursor protein (APP) trafficking and processing underlies the most prominent hallmark of disease in AD affected areas, represented by an abundance of Aβ soluble oligomers, fibrils and plaques. Non-amyloidogenic processing starts with APP shedding by ADAM10, mainly at the cell surface but as well in the trans-Golgi network [10]. Hereafter, the remaining carboxyterminal fragments (CTFs), C83, are processed by γ-secretase to produce harmless p3 peptides and an intracellular domain (inset **a**). In the amyloidogenic pathway (inset **b** for details), which occurs more dominantly in neurons, APP is cleaved first by BACE1 preferentially in endosomes. This endosomal encountering is spatially regulated as BACE1 and APP are found to follow distinct internalization itineraries, the former mediated through ARF6 and GGA3 [11]. C99 is further processed by γ-secretase in recycling and degradative endosomes. It is first cut at position 48 or 49 of the Aβ sequence (ε-cleavage), followed by a carboxypeptidase-like trimming every three amino acids. This results in the production of different Aβ peptides of which the longer ones, like Aβ42 and Aβ43, are more aggregation-prone. γ-Secretase assembly is thought to occur stepwise during ER-Golgi recycling [12]. Assembled PSEN1 complexes (PSEN1/γ) are active at the cell surface and in sorting/recycling endosomes, while PSEN2 complexes (PSEN2/γ) reside in late endosomes/lysosomes due to an aminoterminal sorting motif that selectively binds AP-1. PICALM may mediate internalization of PSEN1/γ, while CD2AP in neurons sorts APP into intraluminal vesicles of MVBs for degradation in lysosomes, instead of through secretase-mediated processing pathways. BIN1 intervenes in recycling BACE1 to the cell surface as well as to lysosomes. BACE1 and APP sorting between distinct compartments involves several other sorting proteins and adaptor complexes of which some are depicted in this figure: such as GGA1, SNX17, retromer and SorLA

Both EOAD and LOAD are characterized by the progressive build-up of amyloid β (Aβ) plaques and Tau-composed neurofibrillary tangles in and around neurons. These hallmarks appear relatively late in disease, stirring the debate whether they are a consequence of earlier pathogenic processes. The intracellular accumulation of Aβ peptides, the primary constituents of amyloid plaques, actually precedes plaque formation. Aggregation-prone Aβ42 already accretes in multivesicular bodies (MVBs) in neurons at early preclinical stages [3]; an accumulation that is observed along other endolysosomal abnormalities. These include, but are not limited to, increased Rab GTPase levels that promote more endocytic pathway activity [4], a defective formation of transcytotic Rab11-containing vesicles [5], and lysosomal dysfunctions [6–8]. The endolysosomal abnormalities correlate therefore better with AD-associated early synaptic dysfunctions and cognitive decline as do extracellular plaques [3, 9]. A direct link between Aβ and the cellular endolysosomal trafficking route is found in the processing of Aβ from the amyloid precursor protein (APP) in the associated compartments (Fig. 1). Herein, APP is cleaved sequentially by BACE-1 (β-secretase) and γ-secretase, a tetrameric complex that in humans includes anterior pharynx-defective 1 (APH-1A/1B), presenilin enhancer 2 (PEN2), nicastrin (NCT) and catalytically active presenilin 1 or 2 (PSEN1/2) [13]. Thus, four major γ-secretase complex variants co-exist within cells and tissues [14]. The distinct subcellular localization of secretases has a direct impact on APP processing (Fig. 1). Given the optimal activity of BACE-1 to be in an acidic pH, the so-called amyloidogenic processing is thought to occur mainly in endosomal compartments from where the produced Aβ can be recycled and secreted [11, 15]. Alternatively, APP can be shedded by α-secretase (ADAM10), localized at the cell-surface but also in the trans Golgi network [10], that prohibits the production of Aβ. Part of APP is routed to late endosomes and lysosomes for alternative degradation or for the generation of in particular longer Aβ, creating an intracellular pool of pathology-relevant Aβ [16]. The importance of these intracellular build-ups as incentives for extracellular aggregation is substantiated by the fact that the intracellular Aβ pools re-emerge first after immunotherapy-induced depletion of both extracellular plaques and deposits within the cell [17]. Of note, the intracellular Aβ pool is majorly generated through PSEN2/γ-secretases as these complexes reside in late endosomal/lysosomal compartments ([18], Fig. 1). This preferential localization is mediated through a unique aminoterminal sorting motif in PSEN2, which is absent in PSEN1, that binds the AP-1 adaptor complex in a phosphorylation-dependent manner for sorting of these complexes between the trans-Golgi network and late endosomes/lysosomes [18]. Interestingly, some FAD-PSEN1 mutations shift the localization of γ-secretase from a broad distribution at the cell surface and endosomes to late endosomes/lysosomes, increasing the intracellular Aβ pool and implying distinct mechanisms of FAD-associated mutations in PSEN1 [18]. In most cases, FAD-associated mutations in *APP* and the *PSEN* genes produce more of the longer Aβ peptides, underscoring that it is not an increase of total Aβ but rather a change in the profile of Aβ that is critically associated with disease onset and progression [19].

**Fig. 2:**
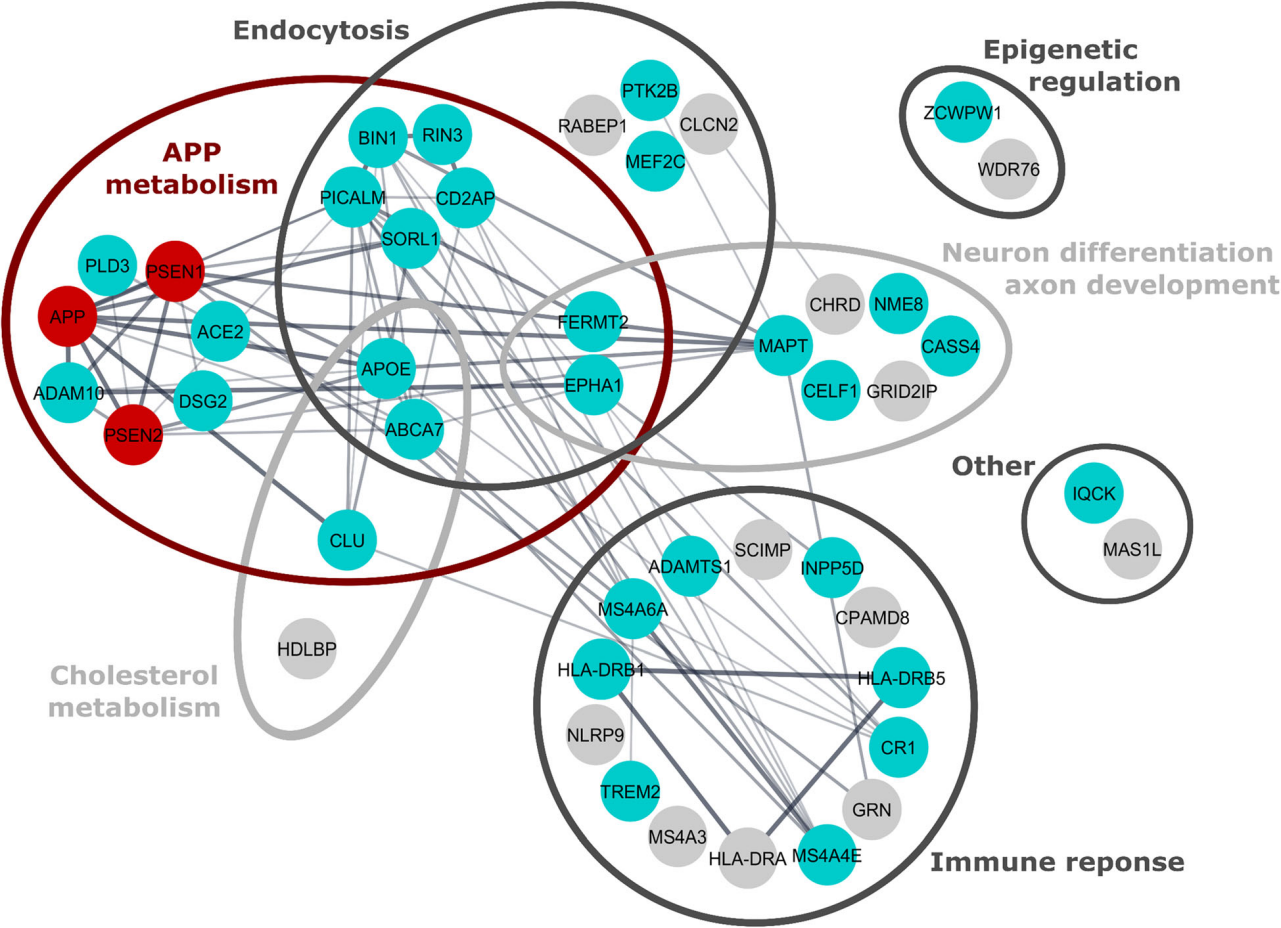
Alzheimer disease risk factors and associated cellular mechanisms. Schematic representation of the biological processes linked to the different proteins being linked to familial AD (red dots) and to an increased risk to develop LOAD (cyan dots). Still to be validated LOAD risk factors are indicated as grey dots. Edges denote protein-protein associations. The figure was generated in Cytoscape with the “String enrichment” plugin and completed with recent interactions from Pubmed articles

**Fig. 3:**
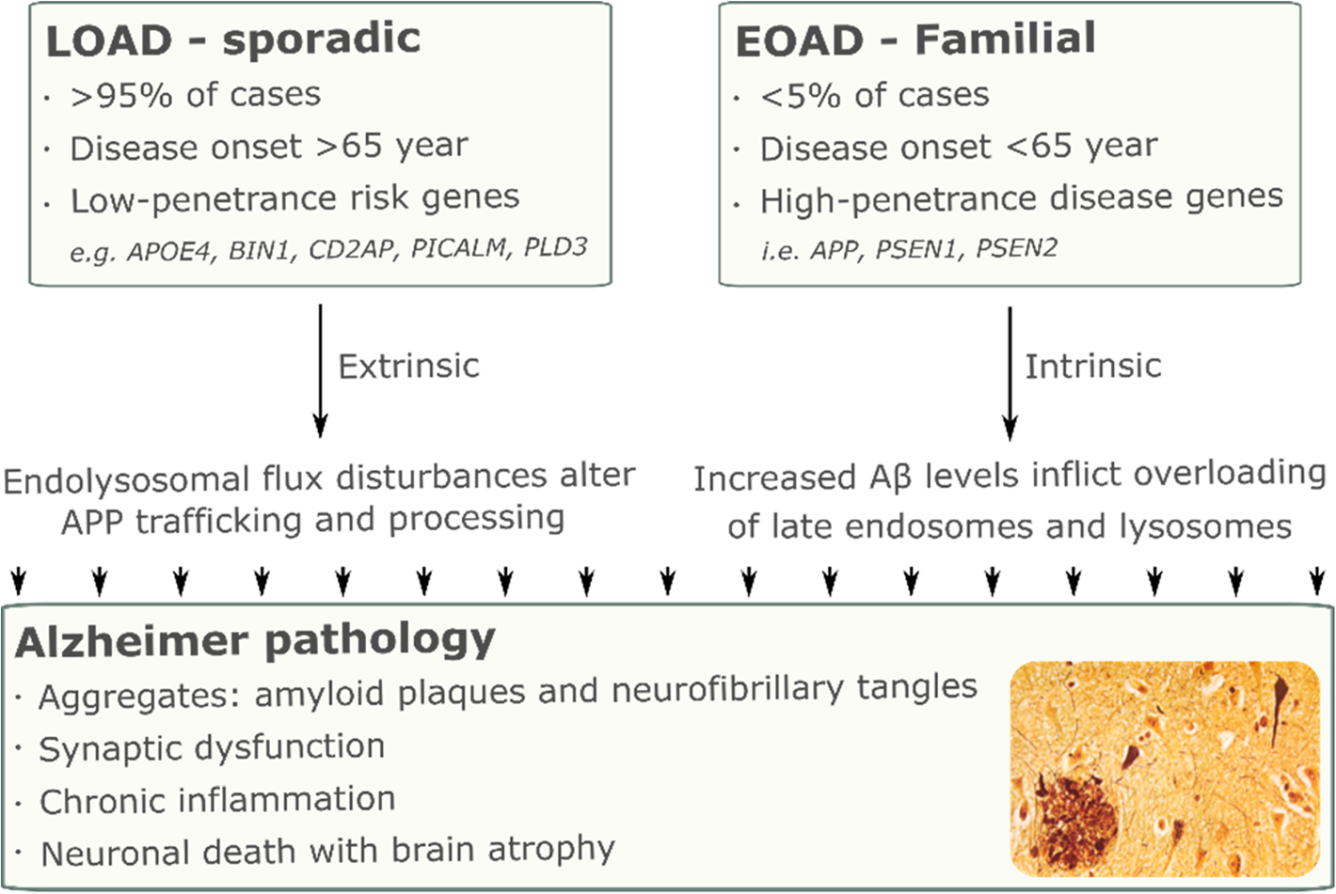
Intrinsic and extrinsic factors dysregulating the endolysosomal flux promote progression to EOAD and LOAD, respectively. APP proteolysis is dysregulated in AD, as shown for instance for familial/early onset AD-associated mutations in *PSEN* genes. FAD-PSEN2 and some -PSEN1 mutations favor a late-endosomal and lysosomal localization of the respective γ-secretase complexes, driving an increased processing of APP into Aβ peptides. Other PSEN1 and PSEN2 FAD mutations shift the intrinsic cleavage functions of the complex, generating more Aβ42 rather than Aβ40. LOAD risk factors, on the other hand, entail the not-optimal working of different cellular mechanisms, e.g. the endolysosomal flux, making dysregulations to be build-up until they also start to impact other mechanisms. Hence, an elevation in the intracellular Aβ pools can originate from two factors: intrinsic ones (mutations linked with APP processing enzymes) and extrinsic ones (variants linked to a dysfunction or lower flux in the endolysosomal system, e.g. trapping γ-secretases in endosomes). While the intrinsic mutations drive FAD/EOAD, the extrinsic variants cause an increased risk of developing LOAD. Both ultimately lead to the characteristic AD clinical phenotype and pathology

To date, (genome-wide) association studies identified 27 AD susceptibility loci: *ABCA7*, *ACE2*, *ADAM10*, *ADAMTS1*, *APOE*, *BIN1*, *CASS4, CELF1, CLU*, *CR1*, *CD2AP*, *DSG2, EPHA1*, *FERMT2, HLA-DRB1, HLA-DRB5, INPP5D, IQCK, MAPT, MEF2C, MS4A6A–MS4A4E, NME8, PICALM, PLD3, PTK2B, SLC24H4-RIN3, SORL1, TREM2* and *ZCWPW1* [20–23]. Though, *MEF2C* and *NME8* did not reach genome-wide significance in every study so far [23]. With new evaluation methodologies and strategies being developed, additional high confidence aspirant genes are being identified including *CHRD*, *CLCN2*, *CPAMD8, GRID2IP*, *GRN*, *HDLBP, HLA-DRA*, *MAS1L*, *MS4A3*, *NLRP9*, *RABEP1*, SCIMP and *WDR76* [24–26]. When grouping these LOAD risk factors into cellular mechanisms it becomes clear that many processes can be directly or indirectly linked to subcellular trafficking routes, in particular related to endolysosomal functioning (Fig. 2). For instance APOE4, ABCA7 and INPP5D are linked to cholesterol transport, while TREM2 and CLU are functionally linked to the clearance of non-native proteins by immune cells. On the other hand, BIN1, CD2AP, PICALM, RIN3, and SORL1 all appear to have functions in endocytic transport regulation, whereas GRN and PLD3 are directed to lysosomes [20–23]. As such, one could argue that a compromised lysosomal homeostasis could be a common denominator in both EOAD and LOAD (Fig. 3). For instance, production and concentration of intracellular toxic Aβ42 species in acidic organelles, as occurs in FAD, could promote Aβ aggregation to higher molecular weight species capable of seeding amyloid fibrils and potentially damaging organellar membranes [27, 28]. Reversely, LOAD associated risk variants may affect the performance and fidelity of endolysosomal transport regulation, impacting on trafficking and localization of APP and/or its secretases as well as hampering the clearance of APP proteolytic fragments and Tau aggregates through a lower flux in lysosomal/autophagic degradation. In the same processes, FAD-mutations are then *intrinsic* factors while LOAD risk genes could be called *extrinsic* factors, affecting the risk of developing AD (Fig. 3).

In this review, we will discuss particularly our current knowledge on the role of LOAD risk factor proteins in endolysosomal transport mechanisms and how this might relate to mechanisms of disease onset and/or progression. Of focus are ‘bridging integrator 1’ (BIN1), ‘CD2 associated protein’ (CD2AP), ‘phosphatidylinositol binding clathrin assembly protein’ (PICALM), and ‘phospholipase D family member 3’ (PLD3). We also include the recent advances on ‘apolipoprotein E’ (ApoE) and ‘triggering receptor expressed on myeloid cells 2’ (TREM2) with emphasis on their role in the endolysosomal system. The reader is directed to Gratuze et al., for a comprehensive review on the role of TREM2 and microglia in the AD pathogenesis [29].

Before going into detail on these LOAD risk factors, it is to be noted that even though there is not always a direct connection with cell- or region-specific expression patterns of (risk factor) proteins and the pathological dysfunctions attributed to them, knowing about endogenous levels, subcellular localisations and disease-linked alterations is key to a better understanding of the protein’s biological functions in health and disease. Additional file 1: Table S1 provides a comprehensive overview on our current knowledge, albeit with the caveat that not all information is human- or nervous system-derived. Moreover, to date, LOAD-risk factors expression and protein levels have been studied globally in LOAD groups or general AD cohorts. It is, however, likely that within a given LOAD risk gene, different single-nucleotide polymorphisms (SNPs) could induce different expression patterns and/or function alterations (Additional file 1: Table S2, Additional file 2: Figure S1). In support of this idea, inducible pluripotent stem cell-derived endothelium from PICALM carriers of the protective rs3851179 SNP shows a ~ 1,7-fold higher PICALM level than those carrying a risk SNP. Homozygous endocytes with the protective PICALM allele also display a twofold higher Aβ40 transcytosis [30]. In addition, the rs10792832 SNP in PICALM has a downregulating effect on the 72.2 kDa variant while leaving the 65.8 kDa form unaltered [31]. SNP-induced transcript alterations are also observed for PLD3 where the synonymous A442A variant (LOAD risk: *p* = 3.78E-7, odds ratio of 2.12) disrupts a splice enhancer binding site, lowering the full length PLD3 mRNA levels in human brain tissue [21]. Hence, caution is needed when interpreting general LOAD cohorts without knowledge of the SNPs in the used population. Aside from SNPs inducing non-sense mutations or splice variations, LOAD risk factor protein expression can be affected by the (LO) AD pathology itself. As an example, PLD3 transcription is altered through AD-linked epigenetic disturbances, highlighting the potential implication of such regulations in the control of LOAD risk factor expression [32]. Hence, even patients without a LOAD risk SNP could be affected by the associated neuronal endolysosomal stress.

## LOAD risk factors in endolysosomal/autophagic functions

### BIN1

BIN1 is a cytoplasmic, vesicle-mediated transport adaptor protein that interacts with for instance Tau, dynamin and clathrin. The major fraction of BIN1 transcripts encodes low molecular weight (~ 65–75 kDa) isoforms, which are primarily associated with mature oligodendrocytes. Neurons express full length, exon 7-containing BIN1 (90 kDa), but at a ~ 4-fold lower level; a ratio which is further tilted in favour of the shorter variants when assessing AD brains [33, 34]. On the subcellular level, BIN1 proteins are broadly distributed across endosomal structures, including early to late endosomes, lysosomes and recycling endosomes [35, 36]. When not localized to synaptic terminals [37], BIN1 can be detected at axonal initial segments and at nodes of Ranvier [38], as well as along axonal Tau neurofilaments [33, 34]. BIN1 is indeed involved in polarized (axonal) endolysosomal transport and amyloidogenic processing herein. In primary hippocampal neurons, BIN1 controls the generation of Aβ through the regulation of APP and BACE1 endocytic trafficking [39]. BIN1 is predominantly active in early axonal endosomes where it enables tubule scission and subsequent BACE1 recycling to the plasma membrane [39]. In addition, BIN1 is involved in BACE1- and APP-trafficking to the lysosomes in an ubiquitin-independent and -dependent mechanism, respectively [39, 40]. However, this role could not be recapitulated in vivo; mice bearing a single *BIN1* allele deletion show no alterations in endosomal sorting of BACE1 and, hence, no impact on the C-terminal fragment levels of APP that result from BACE1 cleavage (Fig. 1, [41]). While these results require further investigation, BIN1 has also been found implicated in the formation of Tau tangles.

**Fig. 4:**
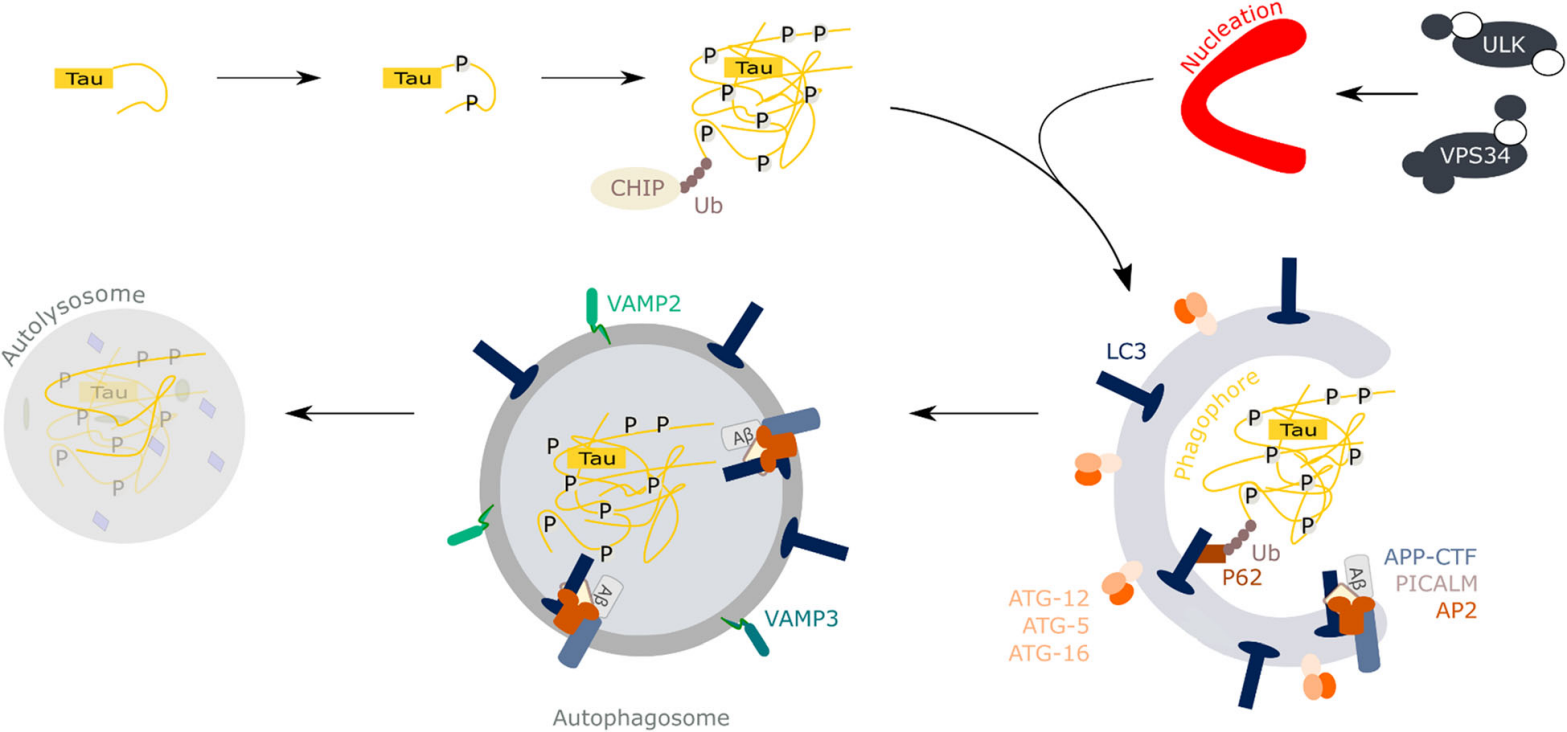
Role of PICALM in autophagy and the degradation of tau fibrils. The catabolic autophagy cascade engulfs cytoplasmic content such as dysfunctional organelles, aggregated proteins or peptides for degradation in lysosomes. Autophagy can be initiated through different upstream (metabolic) signals that converge into the activation of two protein complexes, the ULK complex (ATG13, ATG101, FIP200 and ULK1) and the VPS34 complex (Atg6, Atg14, VPS15 and VPS34). One of the first steps is the formation of the phagophore that recruits ubiquitinated cargo through receptor proteins like p62 as well as LC3. After closure, a double-membrane autophagosome is formed that next can fuse with lysosomes to form autophagolysosomes: this fusion provides the acidic environment and hydrolases to degrade engulfed cellular material. In (LO)AD, Tau gets hyperphosphorylated, which promotes its aggregation into fibrils that are targeted by the cell for autophagic breakdown. The pathway requires Tau to be ubiquitinated by the CHIP (carboxyl terminus of the Hsc70-interacting protein)-Hsc70 complex. Under normal circumstances autophagy can keep pace with the breakdown of Tau aggregates. When this process gets impaired, as occurs in the AD pathology, Tau aggregates cannot be cleared and start to build up, resulting in the formation of neurofibrillary tangles in the end. PICALM dysregulation in LOAD could affect its normal functioning; the interaction and endocytosis of SNARE proteins by PICALM, e.g. VAMP2, VAMP3 and VAMP8, regulates the autophagy process and, hence, removal of Tau. Moreover, through PICALM’s interaction with the complex adaptor protein 2 (AP2), it cross-links LC3 to the APP C-terminal fragment that makes the fragment to be taken up and broken down in the autolysosome

Under healthy conditions, an excess of Tau can be degraded by either the proteasome or the autophagy pathway (Fig. 4), depending on its structure and post-translational modifications. One of the early problems in AD is that the autophagy process, which is most suited to degrade bulk amounts of old and dysfunctional proteins and organelles, is impaired. These defects precede the build-up of neurofibrillary tangles [42]. More specifically, in the case of LOAD, the dysfunctions can be rooted in risk factor proteins impeding endosomal trafficking (as BIN1 [9]), reducing the degradative potential of lysosomes (like PLD3 [43]), abrogating the initiation of autophagy (like PICALM [44, 45]) or generating physical damage to the vesicles (like APOE4 [46–48]). BIN1 does not only directly bind Tau [34, 49], its protein levels inversely correlate with Tau pathology propagation in an in vitro model of *BIN1* knockout rat hippocampal neurons in co-culture with Tau(P301L)-HEK293 cells [37]. The underlying mechanism is found in BIN1 negatively regulating the endocytic flux through Rab5 activation and through its clathrin-interacting CLAP domain. In case of lower BIN1 levels, accumulated Tau promotes new intracellular aggregates by disrupting endomembranes and escaping into the cytosol [37]. The presence of enlarged Rab5-positive endosomes and a hampering of endosomal trafficking will act as an incentive for further cellular debilitating events, including pathogenic amyloid processing and disease propagation [9]. Of note, in the context of AD, it was shown that disease propagation is at least partially based on exo- and endocytosis of seeding oligomeric species of protein Tau and Aβ between neighboring neurons. One of the major routes is through release of exosomes, that equal intraluminal vesicles of multivesicular bodies, and that have been shown to sequester and spread pathogenic protein seeds [50, 51]. Hence, distorted endosomal functioning could affect both normal neuronal communication through neurotransmission as well as actively promote disease spreading.

As mentioned previously, BIN1 binds actin, stabilizing Tau-actin bundles and promoting actin polymerization [52]. In this function, BIN1 can also form dynamic plasma membrane microdomains in mammalian adult ventricular cardiomyocytes. These microfolds containing cytoplasmic BIN1 can be released to generate cell-cell communication through microvesicles. BIN1 enables such a process by recruitment of the ESCRT-III subunit ‘charged multivesicular body protein 4B’ to its BAR domain [53]. Being detected in cardiomyocytes, a similar mechanism is still to be identified in brain-derived cells. However, these BIN1-containing T-tubule domains are not best known for microvesicle generation, but rather for being a calcium signalling apparatus. Interestingly, BIN1 was already found to bind calmodulin in mouse brain lysates [54]. Hence, a role for cerebral BIN1 in both microvesicle formation and/or calcium homeostasis is not to be ruled out. The latter would be of interest regarding the calcium hypothesis of AD, in which calcium alterations may contribute to lysosomal dysfunctions as well as synaptic deficits [55]. Central to the AD pathology lies such a dysfunctional synaptic transmission with a widespread presynaptic cholinergic denervation as a consequence [56]. The connection with the LOAD risk factor BIN1 is found in its binding to dynamin in a 0.5–1:1 ratio. Through the ensuing GTP-dependent membrane fission event, the BIN1-dynamin interaction is able to regulate sizes of synaptic vesicles [57, 58]. Hence, when distorted, endosomal-localised BIN1 could affect not only endocytosis-linked processing of APP, but also the degradation of Tau through autophagy, Tau’s spreading in exosomes and generation of synaptic vesicles.

### CD2AP

CD2AP is a scaffolding molecule functioning in synapse formation through interactions with other adaptors like endophilin, as well as in endocytosis and vesicle trafficking. The 71 kDa protein is strongly expressed in both neurons and blood vessel epithelia (blood brain barrier) [59, 60]. Data on level fluctuations in LOAD patients are awaited. Accordingly to its functions, CD2AP can be found at F-actin^+^/tubulin^−^ neurite tips, branch points and swellings [59]. Importantly, CD2AP also co-distributes with neuronal Rab5 (an early endosomal GTPase) but with a higher preference towards dendrites compared to axons, associating its function more pronouncedly with dendritic endolysosomal flux [39, 59]. When depleted from CD2AP, neurons show an abrogated delivery of endosomal content towards lysosomes in the cell body, including APP. Indeed, CD2AP appears to contribute to the sorting of APP from the limiting endosomal membrane to the lumen for subsequent lysosomal targeting [39]. Hence, knockdown of CD2AP results in endosomal accumulation of APP, promoting its amyloidogenic processing. Neuroblastoma N2a-APP_695_ cells wherein CD2AP is stably knocked down as well as CD2AP^−/−^ primary mouse cortical neurons secrete significantly less Aβ40 [39, 61]. Knocking out CD2AP in PS1xAPP_695_ mice also reduces the Aβ42/Aβ40 ratio of secreted fibrils by 1.3-fold, but no effect is detected on absolute Aβ40 and Aβ42 levels. As the decrease in the ratio occurs before plaque deposition, it is hypothesized that lowered CD2AP levels also impact γ-secretase distribution and/or affect the trafficking of APP to the cell surface [61]. Of note, apart from APP and secretases, CD2AP impacts epidermal growth factor receptor endocytosis and trafficking [62, 63], besides binding and affecting other proteins of de endocytotic machinery (Additional file 1: Table S3.).

CD2AP interacts with the curvature-promoting protein endophilin in HeLa, HEK293T cells, MDCK (NBL-2) kidney cells and drosophila cells [63–65]. In the latter, the interaction has been found essential for the PSEN-mediated processing of the APP-like protein, the Drosophila orthologue of mammalian APP [64, 65]. In human cells, this interaction implicates CD2AP in clathrin-induced endocytosis of synaptic vesicle membranes [63]. Aside from the reuptake of synaptic vesicles, CD2AP is involved in the formation and loading of exosomes through binding the ESCRT component ALG-2-interacting protein X (ALIX) [62]). CD2AP depletion causes the exosomal cargo protein GPRC5B to amass in the vicinity of the cell surface instead of in perinuclear punctate structures, resulting in exosomal GPRC5B levels being reduced by half in HEK293 cells [66]. Whether these defects would affect Tau pathology and how has not been reported. One study detected CD2AP to be a susceptibility locus for cerebrospinal fluid Tau biomarkers [67], though the exact mechanism remains to be determined.

### PICALM

PICALM functions as a clathrin-assembly protein, which recruits the adaptor complex 2 (AP2) to clathrin-coated pits to control membrane cycling. As for CD2AP, endothelial cells prominently express PICALM aside from neurons [68]. Transcript and protein levels have been shown to be both up- and downregulated in LOAD, depending on the single-nucleotide polymorphism (SNP) variant studied (Additional file 1: Table S2, [30]). Eventual loss of PICALM caused by risk SNPs could be exacerbated by an abnormal cleavage of PICALM by calpain and caspase-3, creating 25 and 50 kDa fragments as assessed in human brain extracts and neuroblastoma cells [69]. In line with its functions, PICALM localizes to clathrin-coated pits or vesicles within HeLa cells [70, 71], COS-1 cells [71], and the CA1 hippocampal region/stratum radiatum of rats [72, 73]. Here, and in contrast to CD2AP’s more polarized localization, PICALM is equally spread across synaptic junctions [73, 74]. The interaction with clathrin-coated vesicles is expected to be transient with ~ 40% of PICALM proteins to be found in related compartments such as early and sorting endosomes and tubulovesicular Golgi structures near dendritic branch points [71, 72]. PICALM is recruited to the cell surface during clathrin-coated pit formation and altering its expression affects local clathrin distribution [31, 70, 71]. For instance, following knockdown, clathrin-coated structures form larger, wide-necked pits. Mini coats appear as well, generating a heterogeneous population in which only 22% of the structures falls in the normal 100–150 nm range [70]. Despite these effects, PICALM generally does not appear to affect the general clathrin-mediated endocytosis uptake of cargo, but only that of specific proteins [75–77], such as vesicle-associated membrane proteins (VAMPs; i.e. VAMP-2, VAMP-3 and VAMP-8), as detected in HeLa and neuroblastoma cell lysates [75, 78]. Through direct interaction, PICALM ensures VAMP internalization and their localization in endosomal compartments where they are required for mediating fusion events [75]. Of the different risk factors, PICALM is most intensively studied with respect to APP proteolysis. For example, the NCT subunit of the γ-secretase complex appears to compete with VAMP8 for binding to PICALM. This is of importance as the NCT-PICALM interaction enables the internalization of γ-secretase at the cell surface, impacting on the endosomal maturation of γ-secretase [76, 78]. As a consequence, non-amyloidogenic processing is promoted in neuroglioma cells in which PICALM is downregulated, as demonstrated with a 60% decreased soluble APPβ over soluble APPα ratio. More moderate effects are also observed for APP protein levels that reach only 73% of control levels in these cells [31]. A decreased amyloidogenic processing in PICALM downregulated cells is also supported in in vivo models. Heterozygous PICALM^+/−^ mice of 5 months old have a 20% reduced Aβ42/total Aβ ratio [78]. When crossed with mice overexpressing human APP, harbouring the Swedish and Austrian FAD mutation, their piriform cortex shows a lower Aβ plaque burden at 15 months, resulting in less astrogliosis, but with no effects on Tau levels [76]. A similar correlate between decreased PICALM expression and plaque load is observed in hippocampi of a related transgenic APP/PS1 model [79].

However, the effects of PICALM expression on APP processing are not yet clarified as also inverse effects have been documented. For instance, lentiviral overexpression of PICALM in primary rat cortical neurons dose-dependently rescues neurons from the toxicity of extracellular soluble Aβ oligomers, as assayed by the retention of both ATP content and MAP2^+^ count [80]. In addition, APP^sw/0^xPICALM^+/−^ mice of 9 months of age have hippocampal and cortical Aβ loads that are 4-fold higher compared to APP^sw/0^xPICALM^+/+^ mice [30]. This negative correlation between APP pathology and PICALM would be in accordance with PICALM enabling LC3-II to bind to APP-CTFs for autophagic degradation through its interaction with AP2 ([81], Fig. 4). Hence, it is most likely that the overall observed effect can be attributed to the combination of all the mechanisms that are represented by the model system and the specific conditions used. Factors such as expression levels that are influenced by the impact of splice variants and SNPs may affect as well PICALM’s cellular distribution, association with binding partners and as such, its overall fidelity in endocytic transport regulation. In support, depending on the cell model used, PICALM knockdown also differentially affects transferrin endocytosis [31, 70, 77, 79].

As can be inferred from the above, PICALM contributes in the normal process of autophagy (Fig. 4). In HeLa cells, PICALM co-localizes with ATG16L1 on autophagic precursors [45], whereas in frontal cortices of patients with different neurodegenerative diseases, including AD, PICALM levels inversely correlate with those of the autophagic markers LC3-II and Beclin-1 [44]. Hence, when PICALM levels drop below the normal range, this leads to autophagic dysfunctions. The dysregulations already occur in the early steps of the process, with lower numbers of ATG5/ATG12/ATG16L1-positive phagophore precursors being present [45]. Moreover, the ATG12 vesicles that are present, are of a smaller size due to less homo- (VAMP2) and heterotypic fusion (VAMP3) of VAMP proteins. As such, VAMP8-regulated autophagosome-lysosome fusion was also found defective in the absence of PICALM [45]. This PICALM depletion-induced autophagic dysfunction can functionally cross intersect with AD-Tau pathology [74] (Fig. 4). Alternative splicing of PICALM is positively correlated with Tau tangle burden (Braak stage) and is primarily glial in nature [82, 83]. PICALM co-localizes with phosphorylated-Tau (3R and 4R), which exists in an inverse relationship with the levels of the autophagic markers LC3-II and Beclin-1. Of note, this correlation is not only found in homogenates of frontal cortices of AD patients, but of other neurodegenerative diseases as well, e.g. frontotemporal lobar degeneration, Pick Disease and progressive supranuclear palsy [44]. In accordance, knockdown of PICALM in HeLa cells results in a build-up of both Tau and phosphorylated Tau in addition to autophagic substrates such as p62 and mutant huntingtin [45]. Together, such an inverse link with Tauopathies could be obtained through a PICALM depletion-induced autophagic dysfunction [44].

### PLD3

PLD3 is an atypical member of the phospholipase family as its canonical lipase domain (HXKXXXXD motif) has a conserved Asp to Glu substitution, prohibiting the production of phosphatidic acid from phosphatidylcholine [84, 85]. Two transcript variants exist, one of 2200 bp, which is ubiquitously expressed, and one of 1700 bp that is highly expressed in neurons. PLD3 expression is about halved in AD brains and its cellular depletion is further linked to an increased presence in senile plaques [21, 32, 86]. Being lysosomal localized, PLD3 can be found mainly in the soma and proximal neurites of cortical and hippocampal-pyramidal neurons as well as in dentate gyrus granule cells of human non-AD and AD brains [86]. It is a glycosylated type 2 transmembrane protein that during transport through the secretory pathway becomes proteolysed by cysteine proteases, releasing a carboxy-terminal luminal domain fragment, likely the active protein, that finally becomes sorted to lysosomes [87–89]. PLD3, like its closest homologue PLD4, functions as a lysosome-resident exonuclease alike spleen phosphodiesterase, with ssDNA as a substrate [43]. As such, both dendritic cells and macrophages of PLD3-deficient mice do show exaggerated toll-like receptor 9 (TLR9) inflammatory responses, having ssDNA being a ligand of TLR9 [43]. Data on whether PLD3 risk variants would also lead to substrate accumulation and general abrogation of lysosomal functioning as well as whether neurons would encounter similar problems are currently not known.

On the other hand, PLD3 has already been linked to endosome-to-Golgi retrieval [87, 90]. When knocked out in SH-SY5Y and HeLa cells, the formation and stability of tubular structures positive for sorting nexin-1 and MICALL1 are about 40 and 75% lowered, respectively. The levels of APP processing regulator sortilin-1 are further reduced, leading to less sortilin-1 association with APP and, as a consequence, more processing of APP to Aβ [87]. A 50% knockdown in N2a-APP_695_ cells leads to almost a doubling of extracellular Aβ42 and Aβ40 levels, which can be rescued by overexpressing *PLD3* [21]. In contrast however, an in vivo study on 3 months old PLD3^−/−^ mice crossed with the App^*NL-G-F/NL-G-F*^ knock-in model did not detect increased Aβ40 and Aβ42 levels in cortices and hippocampal tissue. Interestingly and reflecting the functional contribution of PLD3 in lysosomal homeostasis, primary and secondary lysosomes had increased densities and dimensions and an almost five times higher prevalence of intra-lysosomal lipid droplets [91]. Hence, it might not be excluded that compensatory mechanisms in vivo could be concealing the impact on APP pathology at early time points in the disease progression.

Thus far we have discussed the implications of LOAD risk factors PICALM, BIN1, CD2AP and PLD3 with respect to their postulated roles in endocytic and lysosomal transport regulation in particular to what is known in neurons and in the brain. However, due to their ubiquitous expression and general functioning in keeping the endolysosomal flux directly or indirectly in check, their altered expression in LOAD cases may have a broader physiological impact relevant for AD neuropathogenesis of which some aspects are discussed hereafter.

## Blood-brain barrier integrity and amyloid transcytosis

**Fig. 5:**
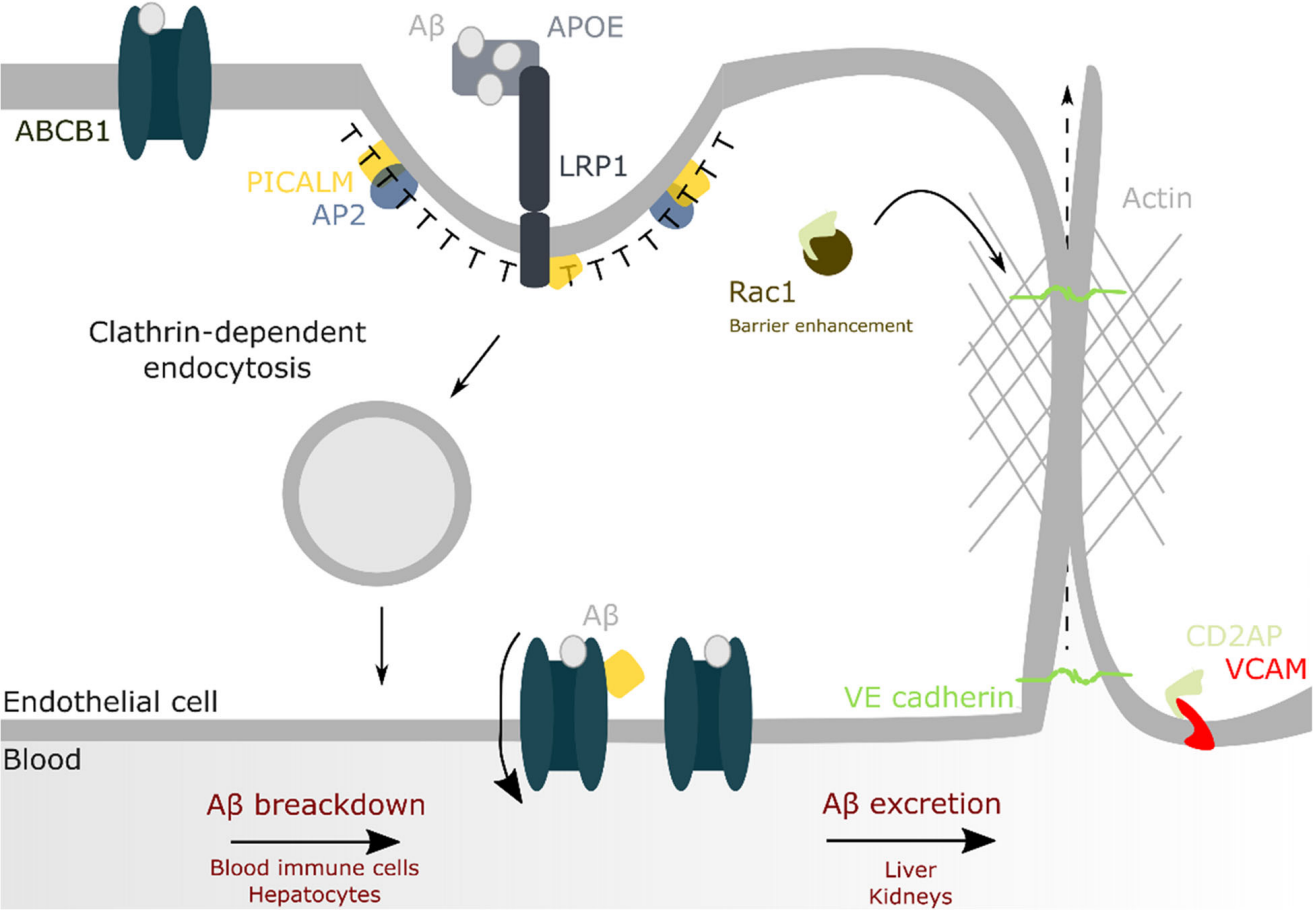
Involvement of LOAD risk factors at the BBB. Transport of Aβ through endothelial transcytosis is PICALM-dependent and a major pathway for clearing Aβ from the brain. Aβ-bound ApoE and free Aβ bind the low density lipoprotein receptor-related protein 1 (LRP1) that associates with PICALM during transcytosis. CD2AP contributes in the upkeep of BBB integrity through a process hypothesized to involve its binding-partner Rac1. Endothelial CD2AP further interacts with VCAM (CD106) and ICAM-1 (CD54), affecting immune cell extravasation

Being a physical barrier between the brain and the peripheral system, the blood-brain barrier (BBB) maintains different transcytosis and paracellular transport mechanisms. One of these pathways enables the clearance of excessive neurotoxic Aβ through peripheral mechanisms ([92], Fig. 5). Although cerebral cells can take up some Aβ for clearance, the periphery is well equipped to remove Aβ from the system, with peripherally-produced or brain-exported Aβ generating no aberrant pathology. If not broken down by blood immune cells and hepatocytes, Aβ can be excreted through the liver or kidneys [93]. Hence, an abrogated export of neurotoxic Aβ from the brain, would contribute to an increased risk in developing AD. PICALM and CD2AP have been shown to be directly or indirectly involved in the process of Aβ BBB transcytosis. The importance of the proper functioning of this pathway is supported by patients harboring SNPs in either of these risk factors to not only present with an increased risk for LOAD, but for vascular dementia as well [94]. Primary endothelial cells of the frontal cortex of AD patients indeed show a ~ 50% reduced basolateral–to–apical transcytosis of Aβ. The same cohort had ~ 34% lowered *PICALM* expression as compared to controls, suggesting a potential critical role for PICALM in this mechanism [30]. This function would be in accordance with (i) the binding of PICALM to the ABC transporter P-glycoprotein and the low density lipoprotein receptor related protein-1 [30, 95], (ii) its expression being primarily linked to CD31/vWF-positive microvessels [96], and (iii) results from heterozygous knockout models (as *PICALM* knockout is embryonically lethal) that show an Aβ40 and -42 efflux across the BBB that is reduced by 41 and 61%, respectively [30]. In support for its critical role in the BBB, endothelial-specific rescue of PICALM expression almost re-normalizes the Aβ load as well as Aβ40 and Aβ42 levels [30]. In contrast to a transcytosis mechanism that gets abrogated, the flow from the interstitial fluid to the cerebrospinal fluid is unaffected by PICALM levels [30]. The latter may be an explanation as to why this route is used as a compensatory rescue one when the PICALM^+/−^ mice are further crossed with APP^*sw/0*^ mice, leading to ~ 2.4/2.5–fold increased interstitial fluid Aβ_40_/_42_ levels at the age of 3 months [30].

The BBB of AD patients does not only display dysfunctional transcytosis, its integrity also gets compromised. The incidence of cerebral microbleeds is about thrice as high in AD patients than in elderlies with a normal cognition [97]. Moreover, brain infarct events are not just significant modifiers of the degree of AD clinical impairments. A multifactorial data-driven analysis showed LOAD patients to exhibit ~ 80% more intra-brain vascular dysregulation as compared to alteration degrees in other mechanisms, including Aβ processing and metabolism [98, 99]. Interestingly, LOAD risk factors have also been connected to BBB integrity. CD2AP^−/−^ mice generally show a renal failure-linked lethality. However, when solved by nephrin-driven *CD2AP* expression, it is possible to detect an increased BBB permeability, which is not associated with proteinuria. In accordance, these mice exhibit an increased susceptibility to seizures [100]. Nonetheless, a barrier failure may not be limited to the BBB. CD2AP is indeed not only expressed in neurons and blood vessel epithelia, but also in lymphocytes, the liver and kidneys [59, 60, 101]; organs known to be important for Aβ peptides degradation and excretion. Hence, aside from compromising the BBB integrity, CD2AP dysregulations could also impact the removal of amyloid peptides through peripheral systems. Reductions in CD2AP levels have already been shown to affect the orientation and positioning of glomerulus cells of the kidneys in vivo [102]. The latter can, at least in part, be attributed to a CD2AP link to Rac1 and its role in cell-cell contacts (Fig. 5): CD2AP is responsible for mediating the interaction between Rac1 and ICAM-1 (intercellular adhesion molecule-1, an integrin binding protein), and possibly VCAM. When impaired due to CD2AP loss, ICAM-1 clusters into complexes, leading to a 5-fold increase in transcellular migration and a reduced lateral migration distance of neutrophils on human umbilical vein endothelial cells [103, 104]. If recreated at the BBB, this would entail an increased influx of inflammatory cells in the brain, potentially contributing to the inflammatory profile of (LO)AD.

Although BIN1 has not yet been implicated in BBB integrity, intestinal barrier tightening has been observed in a mouse model of colonic inflammation treated with a BIN1 monoclonal antibody [105]. Overall, and besides their role in affecting APP processing through endolysosomal regulation, possible dysregulations of paracellular transport mechanisms caused by LOAD risk SNPs should not be ignored, in particular when viewing BBB-crossing pathways as interesting targets to deliver compounds to the central nervous system [106]. If a considerable share of the known LOAD risk factors is indeed acting on BBB functions, this would represent an additional obstacle to get medication into patient’s brains.

## Neuronal outgrowth, connectivity and plasticity

CD2AP is a positive regulator of collateral sprouting, directly impacting neurite length and complexity, and supporting plasticity [59]. In cerebellar granule neurons, it signals through tropomyosin receptor kinase A (TrkA) and p85, and enables outgrowth signals to cross long distances by promoting TrkA incorporation in RAB5^+^ signalling endosomes [59]. Furthermore, PICALM depletion reduces both the number and outgrowth of dendrites in young neurons [72, 107]. Though this phenotype could be attributed in part to a loss of the polarized distribution of VAMP2, VAMP2 mis-sorting on itself is not able to generate the *PICALM* knockdown neurite growth phenotype [72]. Interestingly, overexpression of PICALM in embryonic rat hippocampal neurons promotes the generation of ~ 20% more axons and not at the expense of neurites [72]. Of note, these data demonstrate that LOAD-associated SNPs could modify outgrowth and plasticity mechanisms through affecting the expression and thus normal functioning of the proteins, and which could also underlie a worsened synaptic function and transmission in the neuronal network of AD patients.

## Cell signalling involved in cell cycle regulation and apoptosis

Like most tissues, the nervous system contains stem cells which, through amplification and differentiation, are capable of replacing damaged and/or aged cells. Interestingly, oligomeric Aβ_42_ has been found to possess neurogenic characteristics, a property not shared by monomeric and fibrillary Aβ_42._ Hence, it could be hypothesized that neurogenesis is promoted in the early stages of the disease, while being downregulated in later stages, even further hampering brain homeostasis [108]. In support, while adult hippocampal neurogenesis is abundant in neurologically healthy subjects, it decreases abruptly in AD patients [109]. Though the impact of LOAD risk factors on this matter needs to be studied much more in detail, using (human) neuronal models, other systems already allude to especially CD2AP impacting cell renewal and cell death mechanisms.

A knockout of *CD2AP* in human epithelial cells increases the prevalence of disordered (isotropic) actin structures, leading to dysfunctional cell migration and wound healing characteristics [110]. These results are in line with those of CD2AP^−/−^ HeLa cells that display late defects in cytokinesis, leading to multinucleation being observed [111]. CD2AP actually binds anillin, a protein well known for its role in regulating cytoskeletal dynamics, e.g. as during cytokinesis [111]. Since CD2AP is also attributed with a role in cell differentiation [60], it can be hypothesized that CD2AP-associated risk SNPs oppose cell renewal, putting pressure on homeostatic balance preservation. In support of this idea, downstream of CD2AP, loss in TGF-β signalling results in elevated pro-apoptotic p38-signalling and less anti-apoptotic PI3K/AKT and ERK1/2 pathway activity [112]. An effect can also be expected from the direct interaction between CD2AP and p85, the regulatory subunit of PI3K [64]. As both studies have been performed in glomerular podocytes, the confirmation of this cascade in neuronal cells is still awaited.

BIN1 in turn binds to E3 ubiquitin-protein ligase Itchy homolog (ITCH), which regulates the protein stability of p63 and, as such, both the cell cycle and the apoptosis process [113, 114]. PLD3 is a potential senescence marker, appearing in membranes of p16^+^ or p21^+^ human bladder cancer EJ cells in the senescent stage as assessed in a mass spectrometry screen [115]. A PLD3 knockout in NIH/3T3 and primary mouse fibroblasts promoted resistance to oxidative stress, leading to 10% more cell survival [116]. Given that PLD3 risk SNPs lower full length mRNA levels in LOAD patients, it is to be investigated how this would work and to which variants these apparent opposing effects could be attributed [21]. Lastly, PICALM was detected to play a role in the cellular iron metabolism [117, 118]. PICALM knockout mice do not only display signs of anaemia as cell autonomous erythroid defects and lower haemoglobin levels, they present with a dysregulated haematopoiesis as well, resulting in a defective spleen B cell population and fewer bone marrow cells [117]. The iron deficiency phenotype is accompanied by lower intracellular labile iron pools, limiting cellular proliferation in general [118].

## Neuronal support provided by oligodendroglia

Oligodendroglia are the key iron-containing cells in the brain and, when damaged, may also contribute to detrimental amyloid aggregation through the release of these iron pools [119]. Interestingly, oligodendroglia are the main BIN1 expressing cells of the central nervous system with their BIN1 levels being four times as high compared to neurons [33]. This difference in expression pattern gets even more pronounced during AD progression when oligodendrocytes start to upregulate BIN1 levels while those in neurons get downregulated [33, 34, 120]. Though, it is to be noted that this decrease could be a reflection of the neuronal segment to die off when AD progresses. In addition, it remains to be elucidated whether the BIN1 upregulation would contribute to the protective functioning of oligodendroglia towards neurons, preserving neuronal homeostasis. Sustained high activation grades could also tip the balance into a pathological state, similar as to the one of astroglial scar formation; a general mechanism induced to cope with nearly any type of damage of the brain that flips into a detrimental feature itself. In this regard, mutations in *BIN1* are not restricted to AD with some also being associated to neuromuscular phenotypes [33, 121].

While oligodendroglia have not been of primary focus in the (LO) AD research field, evidence is growing that they contribute to disease progression and the neuronal damage associated with it: (i) neurofibrillary tangle formation in AD is linked to myelination patterns, (ii) hub proteins - such as CNP, PLP1 and MYRF - of the myelin-oligodendrocyte network are strongly dysregulated in AD in vitro and in vivo [122, 123], and (iii) PSEN1 mutations cause oligodendroglia to die at an expedited rate, with their calcium homeostasis being dysregulated and the cells being more vulnerable for the excitatory glutamate and amyloid peptides characteristic to AD [124]. Moreover, oligodendroglia not only support neuronal functioning through generation of neurotrophins and stabilizing neuronal connections, neurons themselves signal through glutaminergic synaptic junctions that are formed with the processes of a subset of oligodendrocytes, which possess calcium permeable AMPA receptors [125]. Hence, either beneficial or detrimental, it is likely that BIN1 dysregulations in one cell type could impact others, based on its associated functions. However, the impact of LOAD BIN1 variants has not been studied in this context so far.

## Alterations of endosomal functions related to ApoE and Trem2

### ApoE beyond cholesterol metabolism implications

ApoE is the best known and, besides ageing, the first discovered risk factor for LOAD [126]. Three ApoE alleles exist, differing only at two residues from one another [ApoE2 (Cys112, Cys158), ApoE3 (Cys112, Arg158), and ApoE4 (Arg112, Arg158)]. Most people are carriers of ApoE2 or ApoE3 alleles, 7 and 79% of the population, respectively. Considerable regional differences are noted for the E4 allele frequency: globally, about 14% of people are estimated to carry at least one ApoE4 allele, with ~ 2% being homozygous carriers (ALZGENE). While the ApoE2 allele reduces the chance to develop LOAD, one copy of the ApoE4 variant elevates the risk for AD up to three-fold, while ApoE4 homozygosity increases this further to a 12 times higher risk [127]. These are population-based values, which may deviate from an individual-level risk one has regarding the cumulative effect of SNPs in a person’s genome [128, 129]. Notwithstanding, the ApoE2 allele keeps getting ascribed with a beneficial/protective effect that may in part be attributed to a recently discovered impact of glial-secreted ApoE on the activation of surface ApoE receptors [130]. The subsequent differential downstream induction of the DLK-MKK7-ERK1/2-MAP kinase pathway causes *APP* to be expressed in a ApoE4 > ApoE3 > ApoE2 potency rank order [130].

**Fig. 6:**
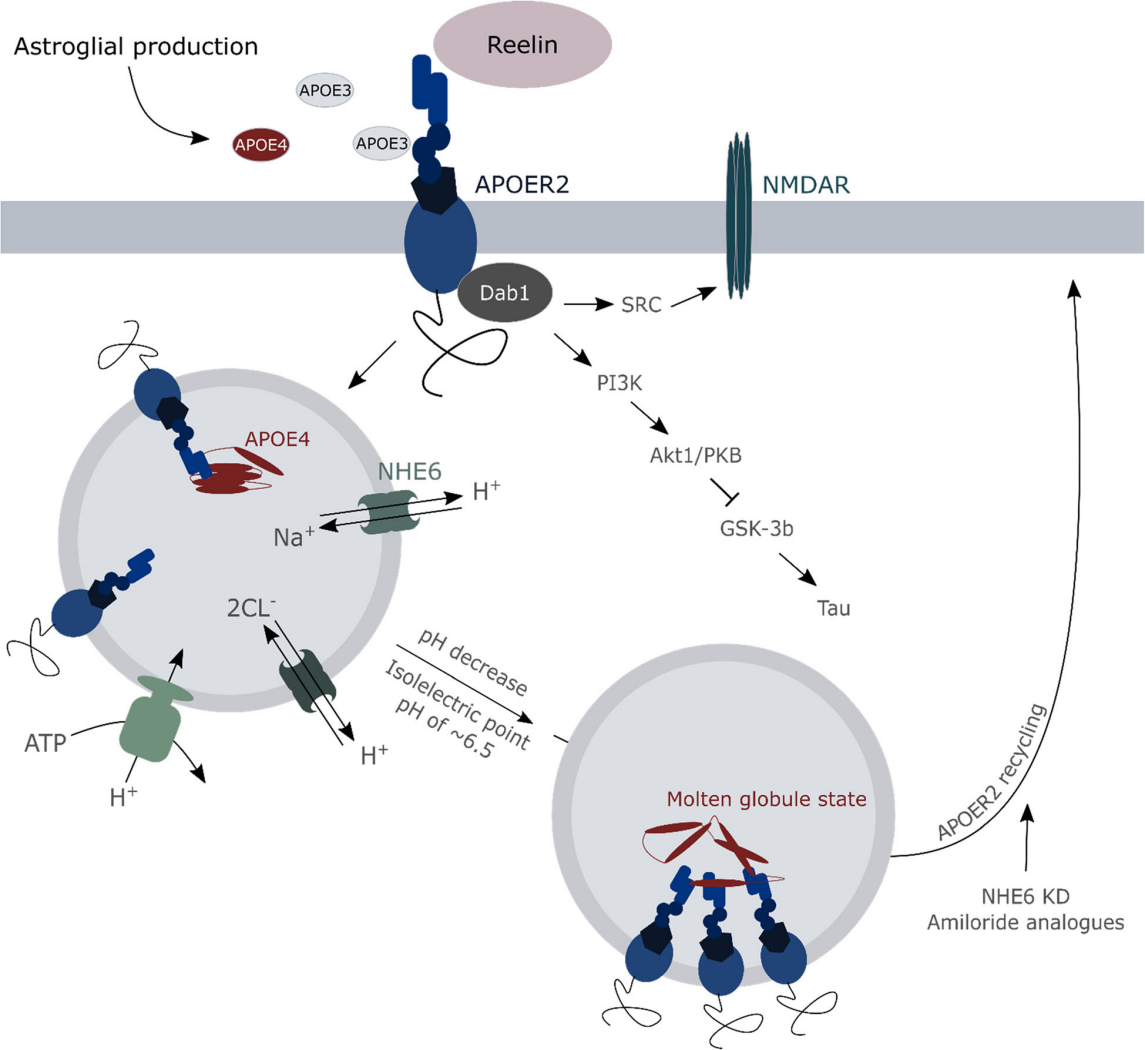
Vesicular acidification by NHE6 impacts ApoE4 stability. The APOE receptor 2 (APOER2) binds the different ApoE isoforms, which are subsequently internalized into endosomes. Passing through the cellular transport axis, endosomes start to acidify through a continuous electrogenic proton influx, pumped into to lumen by V-ATPases. To ascertain that proton accretion is bound within limits, channels can exchange these protons for 2 Cl^−^ or Na^+^, performed by i.a. the Na^+^/H^+^ exchanger NHE6. Under healthy conditions, the acidification enables the APOER2 to be dislodged from APOE, which then relocates to the cell surface again to contribute in N-methyl-D-aspartate receptor (NMDAR) activation. Fluctuations of pH are not only important for a proper receptor recycling, but also for a correct folding of ApoE. The isoelectric point of ApoE4 is higher as the ones of ApoE2 and E3, making its isoelectric point to be already reached in endosomes in contrast to the other variants. Hence, ApoE4 will adapt a molten globule state in sorting endosomes-lysosomes, which not only promotes APOER2 clustering (hindering recycling to the plasma membrane) but also displays a high affinity for phospholipids. These are most likely oxidatively damaged, impacting the mechanical characteristics of the membrane and, as such, their capacity to withstand disruptions e.g. induced by Aβ. The therapeutic potential of targeting this pH-linked instability of ApoE4 has been positively assessed, reducing the levels of the Na^+^/H^+^ exchanger NHE6 and using sodium channel blocking amiloride analogs. Aside from ApoE isoforms, the APOER2 interacts with the extracellular matrix serine protease – Reelin – in a competitive fashion. Upon activation by Reelin, a signaling cascade is induced which abrogates tau hyperphosphorylation and promotes the influx of Ca^2+^ through the NMDAR

Aside from having an impact on *APP* expression, ApoE was found to play an important role in APP metabolism as it is involved in the uptake of extracellular Aβ, through binding the Aβ_12–28_ region to facilitate its clearance from the interstitial space [131]. ApoE2 and ApoE3 are more efficient in performing this function than ApoE4. As a result, double transgenic ApoE4xAPP^SW^/PS1^dE9^ mice show two-fold higher extracellular Aβ loads as do ApoE2 or ApoE3 carriers [131]. The most critical step for correct ApoE functioning in this process appears to be the seeding stage. Amyloid deposition kinetics indeed follow an S-curve in which the nucleation of deposits takes some time (nucleation phase), leading to the generation of small aggregates (seeding phase) on which further deposits can be quickly added (rapid growth phase). In this last phase, the expression of ApoE4 in humanized ApoE mice did not significantly affect the plaques anymore in comparison to those of ApoE3 allele-expressing mice [132]. The underlying reason can (in part) be found in the critical concentration for Aβ deposition into insoluble plaques to be reached faster in APP_SWE_/PS1ΔE9 mice carrying the ApoE4 allele [132]. In addition, Prasad and Rao described a new way of ApoE4 impacting the internalization of Aβ in an astroglia model of human ApoE isoform-expressing ApoE^−/−^ mice [133]. The presence of the ApoE4 variant in astrocytes co-occurs with elevated nuclear levels of the histone deacetylase 4. This increase entails epigenetic alterations being carried out, negatively affecting the expression of the Na^+^/H^+^ exchanger NHE6 that regulates the endosomal pH (Fig. 6). Consequently, endosomes become hyper-acidified resulting in a failure of the Aβ receptor low-density lipoprotein receptor-related protein 1 (LRP1) to be recycled to the plasma membrane, thereby negatively impacting Aβ clearance [133]. Such aberrations could also be underlying the reduced LRP8 receptor recycling by the ApoE4 allele, causing synaptic dysfunctions by reducing LRP8-Reelin interactions that are required for downstream NMDA receptor activation and Ca^2+^ influxes [134]. Both LRP8 and NMDA receptor recycling and their plasma membrane levels can be restored in primary mouse cortical neurons by using amiloride analogues (e.g. EMD87580) and by the genetic downregulation of NHE6, pinpointing altered vesicular acidity to be a main driving factor of ApoE4 trafficking dysregulations ([46], Fig. 6).

However, it is to be noted that ApoE allele variance was not found to impact LRP1 recycling in an in vitro assay on primary rat cortical neurons [46] nor in human ApoE-targeted replacement mice [135]. Thus depending on the model used, additional mechanisms could influence the effect of ApoE biology on receptor recycling. In the study of Zhao et al., ApoE4 was detected to show a higher affinity for insulin receptors than ApoE3, prolonging the stay of the receptors in endosomes and, hence, their recycling to the plasma membrane [135]. By contrast, in the model of Xian et al, no effect of ApoE variants was seen on endocytic cell surface receptors that do not directly interact with ApoE itself, e.g. the insulin and transferrin receptors [46]. Irrespective of some discrepancies between studies, aforementioned results point to receptor trafficking being one part of a larger series of vesicular dysfunctions linked to ApoE4. For instance, one ApoE4 allele was found to affect the expression of other endosomal transport regulators (e.g. Rab5b, Rab7 and Snx3) and to result in a higher amount of Rab5a positive early endosomes and lysosomes [136]. ApoE4 carriers exhibit higher levels of the phosphoinositol phosphatase synaptojanin 1 as well [137], which is involved in the maturation of autophagosomes and endolysosomal trafficking in general [138]. Hence, the more pronounced autophagic dysfunction in AD-related ApoE4 carriers could also be attributed to ApoE4 dysregulating the autophagy mechanism.

ApoE4 and dysregulated lysosomal acidification are, in general, firmly connected. RNA-seq data from ApoE3/4 mouse entorhinal cortices reveal an upregulation of the expression of up to nine subunits of the endolysosomal V-ATPase (proton pump) as compared to ApoE3/3-linked levels [136]. Moreover, ApoE4 has already been described with different deleterious effects when localized in an acidic environment [47, 48, 139]. Structural lability of ApoE4, due to a higher isoelectric point than the other ApoE isoforms, makes it adopt a molten-globule state in acidic environments that is prone to promote receptor clustering ([46, 140, 141], Fig. 6). ApoE4 reactively binds phospholipids and destabilizes the membrane, causing disruptions with lysosomal leakage as a consequence [47, 48]. Because of its reduced solubility, ApoE4 is more susceptible to accumulate in lysosomes when cellular lipid peroxidation levels are high and in the presence of an iron overload [142], which are characteristics of AD-linked ferroptotic cell death. These effects could be reinforced by astroglial secreted ApoE (both ApoE3 and ApoE4) enhancing the lysosomal pathway [48].

ApoE4 may further impact another clearance pathway of Aβ, namely transcytosis through the BBB. It was shown that pericytes of the BBB are able to clear Aβ42 aggregates in a LRP1/ApoE isoform-specific interaction, with the mechanism only working with astrocyte-derived lipidated ApoE3 but not with ApoE4 [143]. It has not yet been checked whether ApoE2 would be even more efficient in the process as is ApoE3. Aside from an increased concentration-based likelihood to form oligomers, it is not to be overlooked that ApoE drives Aβ seeding through their binding of pyroglutamylated Aβ fibrils [144].

### TREM2 beyond inflammation suppression

TREM2 is a type I transmembrane glycoprotein of the immunoglobulin-lectin-like superfamily primarily expressed on immune cells. As such, TREM2 has been classified as an inflammation-linked risk factor for LOAD. The most common SNP rs75932628-T (R47H) has an odds ratio of ~ 3 and is associated with a loss of TREM2 functioning [145]. Other variants of TREM2 include Q33X, R52H, R62H, R62C, T66 M, D87N, T96K, R136W, E151K, H157Y, and L211P ([146], Additional file 2: Figure S1). TREM2 elicits (chronic) inflammatory responses in dendritic cells, macrophages and microglia. In HEK293T cells, this inflammatory dysregulation is closely connected to TREM2-linked autophagic functions and with its continuous shuttling to and from the plasma membrane, where TREM2 is shed by ADAM10 [147]. Soluble TREM2 interacts with Aβ42 oligomers and although this interaction is not affected by TREM2 LOAD risk variants, TREM2 SNPs negatively affect Aβ internalization, possibly through affected ApoE interaction [148]. Human induced pluripotent stem cell-derived monocytes and transdifferentiated microglia-like cells with lowered TREM2 expression or TREM2 loss-of-function show a similar impaired clearance of amyloid plaques in an *ex vivo* amyloid plaque clearance assay, comprising sections of mouse brains (6-month-old APP/PS1^+/−^) [149]. This could not only be linked to a reduced Aβ uptake, but with degradation as well. TREM2 can be detected in early and late endosomes [150]. Interestingly, recombinant TREM2 binds to ApoE [151], supporting that TREM2 affects endo-lysosomal functioning reminiscent of ApoE. The TREM2-APOE pathway is actually a major promotor of the microglial phenotypic switch to an amoeboid-phagocytic phenotype in which the tolerogenic characteristics of microglia are subdued [152]. The TREM2-APOE cascade involves miR-155 activation with downstream effectors as Mef2a and PU.1. When activation is lost due to TREM2 deficiency, microglia are stuck in the homeostatic phenotype status, making them unable to respond appropriately towards apoptotic neurons under stress [152].

Dysregulations of TREM2 in AD can also be connected to the mTOR pathway. Anomalous mTOR signalling results in less activation of mTORC1 and mTORC2 effectors. In turn, decreased downstream signalling increases the formation of autophagic vesicles, a morphological characteristic observed in AD patients with TREM2 risk variants and transgenic animals with lowered TREM2 levels [153]. Related to this, decreased levels of the autophagy-specific PI3K complex proteins, beclin1 and Vps34, disrupt retromer-mediated recycling of TREM2 [154]. Interestingly in this regard, brains of general AD post-mortem cohorts also exhibit significantly lowered levels of this complex [154]. Hence, TREM2-linked autophagic dysregulations could be occurring in patients without TREM2 risk SNPs as well.

## Conclusions

In the past decade, genetic meta-analyses provided us with a growing list of risk loci that has led to the identification of risk genes (albeit, for many risk loci the actual gene variant still needs to be identified). In contrast to EOAD risk genes, these risk variants are more commonly found in the population but confer very little overall risk and thus less predictive value regarding the chance of developing AD. However, they allow the identification of enriched pathways that guide molecular cell biologists and biochemists to a better understanding of the cellular dysregulations that initiate a pathological AD cascade. As such it will be of interest to identify those risk factors active in the same pathway or mechanism, and possibly pinpoint a converging point at which different upstream dysregulations tip the balance towards disease [9]. Risk factors active in cholesterol, immunity and endolysosomal mechanisms remain of major interest as these can modulate both Aβ production and clearance events, the latter through activated microglia, the BBB and/or peripheral clearance systems. With further genome analyses being carried out, new LOAD risk factors are expected to be identified, which could further converge on similar affected regulatory pathways. Indeed, a recent study identified new genome-wide loci that suggest or confirm associations with ADAM10, ADAM10TS1 and ACE, demonstrating that genetic variants affecting APP metabolism are also associated with LOAD and not only with EOAD [23]. These genetic analyses will need to be merged into a larger integrative approach comprising the analysis of expression- and epigenetic profiles. As such, the transcription factor PU.1 of the risk factor *SPI1/CELF1* locus was detected as the gene within this locus with the highest association with AD age of onset and to have cis-regulatory, epigenetic-regulated elements in genes as CD33, MS4A4A, and TREM2, explaining the strong link between *SPI1/*PU.1, myeloid cells and AD [155]. Moreover, the study detected expression quantitative trait loci (eQTLs) evaluations of age of onset-defined survival to show less associations if whole brain lysates where used instead of using data from only myeloid cells. Not surprisingly as they make up only a small fraction of the total brain population of cells [155]. These results are in line with the general trend of studying only one specific cell type up to single cells.

Looking at the different functions of risk factors, it may be clear that several are (in)directly linked to the proper functioning of the blood-brain barrier and in particular in transcytotic events. If these mechanisms gradually fail, it will directly affect the efficiency of Aβ clearance to the blood stream. Hence, LOAD patients with such SNPs could present with even more cerebrovascular dysregulations as patients in the general AD cohort [156]. However, the reverse is also true as an optimally working BBB is essential for efficient drug delivery strategies, e.g. currently focused on bifunctional antibodies directed against the transferrin receptor [157]. Thus, the involved pathways should be rigorously tested in a LOAD risk gene background for a drug delivery agent to become successful. This may also hold true for nanomedicines for nose-to-brain delivery [158]. Although the olfactory neuroepithelium is the only region of the central nervous system that is not shielded by the blood-brain barrier, drug-nanocarriers need to reach the brain through intracellular axonal transport across olfactory and trigeminal nerves or uptake in the cerebrospinal fluid; mechanisms that also demand an intact functioning endolysosomal transport system.

Though many risk genes and the proteins they encode function in conserved cellular mechanisms, their expression patterns differ between the different cell types in the brain. For instance, the more pronounced expression of risk factors like TREM2 and ApoE4 have channelled much attention to disease associated microglia (DAMs). It will be a major next task for geneticists to correlate genome-wide associations of risk loci with single cell analyses to identify cell populations that are particularly enriched for the identified risk variants as this will instruct life scientists on what cell types to prioritize when studying the role of these associated risk genes/proteins. A more difficult factor to analyse is when risk variants start to impede on these enriched pathways, leading to AD pathology. As an example, DAMs surrounding plaques could be considered a relatively late event. Thus studying the impact of risk variants like TREM2 or ApoE4 in vivo at the stage of DAMs might not provide us full insights of their contribution to early, preclinical stages of AD pathology, i.e. before plaques start to occur. Further to this, a human-specific context is a critical aspect in deciphering AD relevant molecular mechanisms as evidenced by a human/mouse brain chimeric model [159]. When human pluripotent stem cell-derived cortical neuronal progenitor cells were transplanted in an APP_swe_xPSEN1_L166P_ transgene model, they became integrated into the mouse brain, getting exposed to a high burden of Aβ. This resulted in the human specific pathological features including neurodegeneration, cell death and tau pathology that lack in otherwise classical murine AD models [159]. This transplant model can now be exploited to systematically evaluate the effects of LOAD risk variants, not limited to human neurons but also other cell types such as microglia. Still, many functional aspects of risk gene variants will continue to be studied in cellular models. However, also here, a transition should be made from the classical (non) neuronal *APP/PSEN1* overexpressing cells to patient-derived human neurons, including gene-corrected isogenic controls. In order to cross compare data from different labs, one should put efforts in standardizing differentiation protocols as well as set up functional criteria that should be minimally explored.

Several risk variants described in this review function along spatially distinct steps of for instance endolysosomal regulation; nevertheless they all contribute to the risk of developing AD, indicating that they converge on similar or identical pathways or organellar functions. Thus, besides exploring how variants affect the intrinsic functions of the associated risk gene/protein, their impact on subcellular signalling, metabolomic pathways - including in particular lipid alterations - should not be ignored. One strategy to explore this is to exploit omics profiling of intracellular organelles in a disease context [160]. Plasma membrane omics could provide mechanistic insights in underlying altered endosomal transport regulation, as demonstrated in PSEN deficient cells [161], but also provide novel therapeutic targets as currently about two thirds of known drugs target cell surface localized proteins. Secondly, given the emerging role of lysosomal dysfunction in many neurodegenerative diseases, knowing their biomolecular composition could provide valuable insights on the overall effect that risk variants have on this degradative hub of the cell/neuron. This has been recently validated in Nieman-Pick disease type C1 deficient cells using magnetically isolated lysosomes [162]. These strategies can even be extended to metabolome profiling of lysosomes [163], which bears great promise for applications in the context of neurodegeneration.

While socioeconomic, cultural and environmental factors can differently contribute to the risk for AD, it is also unlikely in most if not all cases that a single LOAD risk SNP may be sufficient to drive progression to AD pathology. Such an oligogenic basis of disease has already been described in patients presenting with amyotrophic lateral sclerosis [164, 165]. In addition, the loss of VPS13C functioning in early-onset forms of Parkinson disease was recently detected to aggravate PINK1/Parkin-dependent mitophagy [166]. Regarding AD, Bonvicini et al., found that 34% of LOAD patients carries 2 or more rare/common risk variants, a percentage that is increased to 69% in the EOAD group [167]. Hence, one could argue that in future genetic analyses, more cases will emerge with combined risk variants. As such, it could also be interesting to look at risk factors' synergistic effects to elucidate LOAD-driving pathways. Finding the true interaction potential of different risk factor SNPs could be applicable across the whole AD spectrum. Epidemiology studies already indicate that correlations exist between a patient’s genetic risk score and endophenotypes [129], making it interesting to start generating subgroups that share phenotypic features and biochemical or cellular pathways. Such information is critical for precision medicine which provides a basis for later personalized medicine.

In conclusion, the plethora of LOAD risk loci and variants underscore the multifactorial nature of AD onset and progression involving the different cell types in the brain and periphery. Deciphering the spatial and temporal functions and defects will however be inevitable to identify the vulnerable pathways and networks that become primarily under pressure in the ageing brain, and that, when traversing a critical threshold, initiate an irreversible and progressive neurodegenerative cascade towards a pathological AD state.

## Additional files

Additional file 1: **Table S1.** Overview of studies on BIN1, CD2AP, PICALM and PLD3 expression levels. The table summarizes the transcript and protein levels of the LOAD risk genes, as detected in in vitro and in vivo studies, as well as the subcellular localization of the associated protein when studied. N.d.; not determined. **Table S.2** LOAD-associated SNPs. Overview of the different SNPs within *BIN1*, *CD2AP*, *PICALM* and *PLD3* that are linked to LOAD development. If studied, the associated clinical effects are listed. **Table S3.** Summary of the known protein interactors of BIN1, CD2AP, PICALM and PLD3. The table further includes the model(s) in which the interaction was studied, as well as the validation method(s), involved region within the proteins, and observed effects downstream of binding. (DOCX 191 kb)

Additional file 2: **Figure S1.** Schematic representation of BIN1, CD2AP, PICALM, PLD3 and TREM2 structures and the relative positions of known SNPs. Abbreviations: ANTH, AP180 N-terminal homology; BAR, bin-amphiphysin-rvs; CATS, family with sequence similarity 64 member A; CBD, clathrin-binding domain; ENTH, epsin N-terminal homology; FHL2, four and a half LIM domains 2; PDE1, phosphodiesterase type 1; SH3, SRC homology 3; VAMP8, vesicle-associated membrane protein 8. (TIF 1027 kb)

## Abbreviations

AD: Alzheimer’s disease
ALIX: ALG-2-interacting protein X
ApoE: Apolipoprotein E
APOER2: ApoE receptor 2
APP: Amyloid precursor protein
Aβ: Amyloid-β
BBB: Blood-brain barrier
BIN): Bridging integrator 1
CD2AP: CD2 associated protein
DAMs: Disease-associated microglia
EOAD: Early-onset AD
FAD: Familial AD
ITCH: E3 ubiquitin-protein ligase Itchy homolog
LOAD: Late-onset AD
LRP: Lipoprotein receptor-related protein
NCT: Nicastrin
NMDAR: N-methyl-D-aspartate receptor
PICALM: Phosphatidylinositol binding clathrin assembly protein
PLD3: Phospholipase D family member 3
PSEN: Presenilin
RABEP1: Rab GTPase-binding effector protein 1
SNP: Single nucleotide polymorphism
TREM2: Triggering receptor expressed on myeloid cells 2
TrkA: Tropomyosin receptor kinase A
VAMP: Vesicle-associated membrane protein

## References

[1] Cummings J, Ritter A, Zhong K (2018). Clinical trials for disease-modifying therapies in Alzheimer’s disease: a primer, lessons learned, and a blueprint for the future. J Alzheimers Dis.

[2] Cacace R, Sleegers K, Van Broeckhoven C (2016). Molecular genetics of early-onset Alzheimer’s disease revisited. Alzheimers Dement.

[3] Takahashi RH, Milner TA, Li F, Nam EE, Edgar MA, Yamaguchi H (2002). Intraneuronal Alzheimer abeta42 accumulates in multivesicular bodies and is associated with synaptic pathology. Am J Pathol.

[4] Ginsberg SD, Alldred MJ, Counts SE, Cataldo AM, Neve RL, Jiang Y (2010). Microarray analysis of hippocampal CA1 neurons implicates early endosomal dysfunction during Alzheimer’s disease progression. Biol Psychiatry.

[5] Canfield SG, Stebbins MJ, Morales BS, Asai SW, Vatine GD, Svendsen CN (2017). An isogenic blood-brain barrier model comprising brain endothelial cells, astrocytes, and neurons derived from human induced pluripotent stem cells. J Neurochem.

[6] Goetzl EJ, Boxer A, Schwartz JB, Abner EL, Petersen RC, Miller BL (2015). Altered lysosomal proteins in neural-derived plasma exosomes in preclinical Alzheimer disease. Neurology.

[7] Lee J-H, McBrayer MK, Wolfe DM, Haslett LJ, Kumar A, Sato Y (2015). Presenilin 1 maintains lysosomal ca (2+) homeostasis via TRPML1 by regulating vATPase-mediated lysosome acidification. Cell Rep.

[8] Coen K, Flannagan RS, Baron S, Carraro-Lacroix LR, Wang D, Vermeire W (2012). Lysosomal calcium homeostasis defects, not proton pump defects, cause endo-lysosomal dysfunction in PSEN-deficient cells. J Cell Biol.

[9] Peric A, Annaert W (2015). Early etiology of Alzheimer’s disease: tipping the balance toward autophagy or endosomal dysfunction?. Acta Neuropathol.

[10] Tan JZA, Gleeson PA (2019). The *trans* -Golgi network is a major site for α-secretase processing of amyloid precursor protein in primary neurons. JBC.

[11] Sannerud R, Declerck I, Peric A, Raemaekers T, Menendez G, Zhou L (2011). ADP ribosylation factor 6 (ARF6) controls amyloid precursor protein (APP) processing by mediating the endosomal sorting of BACE1. PNAS U S A.

[12] Spasic D, Raemaekers T, Dillen K, Declerck I, Baert V, Serneels L (2007). Rer1p competes with APH-1 for binding to nicastrin and regulates gamma-secretase complex assembly in the early secretory pathway. J Cell Biol.

[13] De Strooper B, Annaert W (2010). Novel research horizons for presenilins and γ-secretases in cell biology and disease. Annu Rev Cell Dev Biol.

[14] Hébert SS, Serneels L, Dejaegere T, Horré K, Dabrowski M, Baert V (2004). Coordinated and widespread expression of gamma-secretase in vivo: evidence for size and molecular heterogeneity. Neurobiol Dis.

[15] Schneider A, Rajendran L, Honsho M, Gralle M, Donnert G, Wouters F (2008). Flotillin-dependent clustering of the amyloid precursor protein regulates its endocytosis and Amyloidogenic processing in neurons. J Neurosci.

[16] Gouras GK, Olsson TT, Hansson O (2015). β-Amyloid peptides and amyloid plaques in Alzheimer’s disease. Neurotherapeutics.

[17] Oddo S, Caccamo A, Smith IF, Green KN, LaFerla FM (2006). A dynamic relationship between intracellular and extracellular pools of Abeta. Am J Pathol.

[18] Sannerud R, Esselens C, Ejsmont P, Mattera R, Rochin L, Tharkeshwar AK (2016). Restricted location of PSEN2/γ-secretase determines substrate specificity and generates an intracellular Aβ pool. Cell.

[19] Szaruga M, Munteanu B, Lismont S, Veugelen S, Horré K, Mercken M (2017). Alzheimer’s-causing mutations shift Aβ length by destabilizing γ-secretase-Aβn interactions. Cell.

[20] Lambert J-C, Ibrahim-Verbaas CA, Harold D, Naj AC, Sims R, Bellenguez C (2013). Meta-analysis of 74,046 individuals identifies 11 new susceptibility loci for Alzheimer’s disease. Nat Genet.

[21] Cruchaga C, Karch CM, Jin SC, Benitez BA, Cai Y, Guerreiro R (2014). Rare coding variants in the phospholipase D3 gene confer risk for Alzheimer’s disease. Nature.

[22] Guerreiro R, Wojtas A, Bras J, Carrasquillo M, Rogaeva E, Majounie E (2013). *TREM2* variants in Alzheimer’s disease. N Engl J Med.

[23] Kunkle BW, Grenier-Boley B, Sims R, Bis JC, Naj AC, Boland A, et al. Meta-analysis of genetic association with diagnosed Alzheimer’s disease identifies novel risk loci and implicates Abeta, Tau, immunity and lipid processing. bioRxiv. 2018:294629. 10.1101/294629.

[24] Fernandez MV, Budde J, Del-Aguila J, Ibanez L, Deming Y, Harari O, et al. Evaluation of gene-based family-based methods to detect novel genes associated with familial late onset Alzheimer disease. Front Neurosci. 2018;12:209. 10.3389/fnins.2018.00209 PMC5893779 29670507

[25] Raghavan Neha S., Brickman Adam M., Andrews Howard, Manly Jennifer J., Schupf Nicole, Lantigua Rafael, Wolock Charles J., Kamalakaran Sitharthan, Petrovski Slave, Tosto Giuseppe, Vardarajan Badri N., Goldstein David B., Mayeux Richard (2018). Whole-exome sequencing in 20,197 persons for rare variants in Alzheimer's disease. Annals of Clinical and Translational Neurology.

[26] Jansen I, Savage J, Watanabe K, Bryois J, Williams D, Steinberg S, et al. Genetic meta-analysis identifies 9 novel loci and functional pathways for Alzheimers disease risk. bioRxiv. 2018:258533. 10.1101/258533.

[27] Zhang R, Hu X, Khant H, Ludtke SJ, Chiu W, Schmid MF (2009). Interprotofilament interactions between Alzheimer’s Abeta1-42 peptides in amyloid fibrils revealed by cryoEM. PNAS U S A.

[28] Friedrich RP, Tepper K, Ronicke R, Soom M, Westermann M, Reymann K (2010). Mechanism of amyloid plaque formation suggests an intracellular basis of a pathogenicity. PNAS.

[29] Gratuze M, Leyns CEG, Holtzman DM (2018). New insights into the role of TREM2 in Alzheimer’s disease. Mol Neurodegener.

[30] Zhao Z, Sagare AP, Ma Q, Halliday MR, Kong P, Kisler K (2015). Central role for PICALM in amyloid-β blood-brain barrier transcytosis and clearance. Nat Neurosci.

[31] Thomas RS, Henson A, Gerrish A, Jones L, Williams J, Kidd EJ (2016). Decreasing the expression of PICALM reduces endocytosis and the activity of β-secretase: implications for Alzheimer’s disease. BMC Neurosci.

[32] Blanco-Luquin I, Altuna M, Sánchez-Ruiz de Gordoa J, Urdánoz-Casado A, Roldán M, Cámara M (2018). PLD3 epigenetic changes in the hippocampus of Alzheimer’s disease. Clin Epigenetics.

[33] De Rossi P, Buggia-Prévot V, Clayton BLL, Vasquez JB, van Sanford C, Andrew RJ (2016). Predominant expression of Alzheimer’s disease-associated BIN1 in mature oligodendrocytes and localization to white matter tracts. Mol Neurodegener.

[34] Chapuis J, Hansmannel F, Gistelinck M, Mounier A, Van Cauwenberghe C, Kolen KV (2013). Increased expression of BIN1 mediates Alzheimer genetic risk by modulating tau pathology. Mol Psychiatry.

[35] Leprince C, Le Scolan E, Meunier B, Fraisier V, Brandon N, De Gunzburg J (2003). Sorting nexin 4 and amphiphysin 2, a new partnership between endocytosis and intracellular trafficking. J Cell Sci.

[36] Nakajo A, Yoshimura S, Togawa H, Kunii M, Iwano T, Izumi A (2016). EHBP1L1 coordinates Rab8 and Bin1 to regulate apical-directed transport in polarized epithelial cells. J Cell Biol.

[37] Calafate S, Flavin W, Verstreken P, Moechars D (2016). Loss of Bin1 promotes the propagation of tau pathology. Cell Rep.

[38] Butler MH, David C, Ochoa GC, Freyberg Z, Daniell L, Grabs D (1997). Amphiphysin II (SH3P9; BIN1), a member of the amphiphysin/Rvs family, is concentrated in the cortical cytomatrix of axon initial segments and nodes of ranvier in brain and around T tubules in skeletal muscle. J Cell Biol.

[39] Ubelmann F, Burrinha T, Salavessa L, Gomes R, Ferreira C, Moreno N (2017). Bin1 and CD2AP polarise the endocytic generation of beta-amyloid. EMBO Rep.

[40] Miyagawa T, Ebinuma I, Morohashi Y, Hori Y, Young Chang M, Hattori H (2016). BIN1 regulates BACE1 intracellular trafficking and amyloid-β production. Hum Mol Genet.

[41] Andrew Robert J., De Rossi Pierre, Nguyen Phuong, Kowalski Haley R., Recupero Aleksandra J., Guerbette Thomas, Krause Sofia V., Rice Richard C., Laury-Kleintop Lisa, Wagner Steven L., Thinakaran Gopal (2019). Reduction of the expression of the late-onset Alzheimer's disease (AD) risk-factor BIN1 does not affect amyloid pathology in an AD mouse model. Journal of Biological Chemistry.

[42] Nixon RA, Yang D-S (2011). Autophagy failure in Alzheimer’s disease--locating the primary defect. Neurobiol Dis.

[43] Gavin AL, Huang D, Huber C, Mårtensson A, Tardif V, Skog PD (2018). PLD3 and PLD4 are single-stranded acid exonucleases that regulate endosomal nucleic-acid sensing. Nat Immunol.

[44] Ando K, Tomimura K, Sazdovitch V, Suain V, Yilmaz Z, Authelet M (2016). Level of PICALM, a key component of clathrin-mediated endocytosis, is correlated with levels of phosphotau and autophagy-related proteins and is associated with tau inclusions in AD, PSP and pick disease. Neurobiol Dis.

[45] Moreau K, Fleming A, Imarisio S, Lopez Ramirez A, Mercer JL, Jimenez-Sanchez M (2014). PICALM modulates autophagy activity and tau accumulation. Nat Commun.

[46] Xian X, Pohlkamp T, Durakoglugil MS, Wong CH, Beck JK, Lane-Donovan C, et al. Reversal of ApoE4 induced recycling block as a novel prevention approach for Alzheimer’s disease. Elife. 2018;7. 10.7554/eLife.40048 PMC6261251 30375977

[47] Ji Z-S, Miranda RD, Newhouse YM, Weisgraber KH, Huang Y, Mahley RW (2002). Apolipoprotein E4 potentiates amyloid beta peptide-induced lysosomal leakage and apoptosis in neuronal cells. JBC.

[48] Ji Z-S, Müllendorff K, Cheng IH, Miranda RD, Huang Y, Mahley RW (2006). Reactivity of apolipoprotein E4 and amyloid beta peptide: lysosomal stability and neurodegeneration. JBC.

[49] Malki I, Cantrelle F-X, Sottejeau Y, Lippens G, Lambert J-C, Landrieu I (2017). Regulation of the interaction between the neuronal BIN1 isoform 1 and tau proteins - role of the SH3 domain. FEBS J.

[50] Asai H, Ikezu S, Tsunoda S, Medalla M, Luebke J, Haydar T (2015). Depletion of microglia and inhibition of exosome synthesis halt tau propagation. Nat Neurosci.

[51] Wu JW, Hussaini SA, Bastille IM, Rodriguez GA, Mrejeru A, Rilett K (2016). Neuronal activity enhances tau propagation and tau pathology in vivo. Nat Neurosci.

[52] Dräger NM, Nachman E, Winterhoff M, Brühmann S, Shah P, Katsinelos T (2017). Bin1 directly remodels actin dynamics through its BAR domain. EMBO Rep.

[53] Xu B, Fu Y, Liu Y, Agvanian S, Wirka RC, Baum R (2017). The ESCRT-III pathway facilitates cardiomyocyte release of cBIN1-containing microparticles. PLoS Biol.

[54] Berggård T, Arrigoni G, Olsson O, Fex M, Linse S, James P (2006). 140 mouse brain proteins identified by Ca2+−calmodulin affinity chromatography and tandem mass spectrometry. J Proteome Res.

[55] Briggs CA, Chakroborty S, Stutzmann GE (2017). Emerging pathways driving early synaptic pathology in Alzheimer’s disease. Biochem Biophys Res Commun.

[56] Hampel H, Mesulam M-M, Cuello AC, Farlow MR, Giacobini E, Grossberg GT (2018). The cholinergic system in the pathophysiology and treatment of Alzheimer’s disease. Brain.

[57] Ramjaun AR, Micheva KD, Bouchelet I, McPherson PS (1997). Identification and characterization of a nerve terminal-enriched amphiphysin isoform. JBC.

[58] Takeda T, Kozai T, Yang H, Ishikuro D, Seyama K, Kumagai Y, et al. Dynamic clustering of dynamin-amphiphysin helices regulates membrane constriction and fission coupled with GTP hydrolysis. Elife. 2018;7. 10.7554/eLife.30246. 10.7554/eLife.30246 PMC5780043 29357276

[59] Harrison BJ, Venkat G, Lamb JL, Hutson TH, Drury C, Rau KK (2016). The adaptor protein CD2AP is a coordinator of neurotrophin signaling-mediated axon arbor plasticity. J Neurosci.

[60] Lehtonen S, Tienari J, Londesborough A, Pirvola U, Ora A, Reima I (2008). CD2-associated protein is widely expressed and differentially regulated during embryonic development. Differentiation.

[61] Liao F, Jiang H, Srivatsan S, Xiao Q, Lefton KB, Yamada K (2015). Effects of CD2-associated protein deficiency on amyloid-β in neuroblastoma cells and in an APP transgenic mouse model. Mol Neurodegener.

[62] Rouka E, Simister PC, Janning M, Kumbrink J, Konstantinou T, Muniz JRC (2015). Differential recognition preferences of the three Src homology 3 (SH3) domains from the adaptor CD2-associated protein (CD2AP) and direct association with Ras and Rab interactor 3 (RIN3). JBC.

[63] Lynch DK, Winata SC, Lyons RJ, Hughes WE, Lehrbach GM, Wasinger V (2003). A Cortactin-CD2-associated protein (CD2AP) complex provides a novel link between epidermal growth factor receptor endocytosis and the actin cytoskeleton. JBC.

[64] Huber TB, Hartleben B, Kim J, Schmidts M, Schermer B, Keil A (2003). Nephrin and CD2AP associate with phosphoinositide 3-OH kinase and stimulate AKT-dependent signaling. Mol Cell Biol.

[65] Singh J, Mlodzik M (2012). Hibris, a Drosophila Nephrin homolog, is required for presenilin-mediated notch and APP-like cleavages. Dev Cell.

[66] Kwon S-H, Oh S, Nacke M, Mostov KE, Lipschutz JH (2016). Adaptor protein CD2AP and L-type lectin LMAN2 regulate exosome cargo protein trafficking through the Golgi complex. JBC.

[67] Ramos de Matos M, Ferreira C, Herukka S-K, Soininen H, Janeiro A, Santana I (2018). Quantitative genetics validates previous genetic variants and identifies novel genetic players influencing Alzheimer’s disease cerebrospinal fluid biomarkers. J Alzheimers Dis.

[68] Baig S, Joseph SA, Tayler H, Abraham R, Owen MJ, Williams J (2010). Distribution and expression of picalm in Alzheimer disease. J Neuropathol Exp Neurol.

[69] Ando K, Brion J-P, Stygelbout V, Suain V, Authelet M, Dedecker R (2013). Clathrin adaptor CALM/PICALM is associated with neurofibrillary tangles and is cleaved in Alzheimer’s brains. Acta Neuropathol.

[70] Meyerholz A, Hinrichsen L, Groos S, Esk P-C, Brandes G, Ungewickell EJ (2005). Effect of clathrin assembly lymphoid myeloid leukemia protein depletion on clathrin coat formation. Traffic.

[71] Tebar F, Bohlander SK, Sorkin A (1999). Clathrin assembly lymphoid myeloid leukemia (CALM) protein: localization in endocytic-coated pits, interactions with clathrin, and the impact of overexpression on clathrin-mediated traffic. Mol Biol Cell.

[72] Bushlin I, Petralia RS, Wu F, Harel A, Mughal MR, Mattson MP (2008). Clathrin assembly protein AP180 and CALM differentially control axogenesis and dendrite outgrowth in embryonic hippocampal neurons. J Neurosci.

[73] Petralia RS, Yao PJ (2007). AP180 and CALM in the developing hippocampus: expression at the nascent synapse and localization to trafficking organelles. J Comp Neurol.

[74] Yao PJ, Petralia RS, Bushlin I, Wang Y, Furukawa K (2005). Synaptic distribution of the endocytic accessory proteins AP180 and CALM. J Comp Neurol.

[75] Miller SE, Sahlender DA, Graham SC, Höning S, Robinson MS, Peden AA (2011). The molecular basis for the endocytosis of small R-SNAREs by the clathrin adaptor CALM. Cell.

[76] Kanatsu K, Hori Y, Takatori S, Watanabe T, Iwatsubo T, Tomita T (2016). Partial loss of CALM function reduces Aβ42 production and amyloid deposition in vivo. Hum Mol Genet.

[77] Harel A, Wu F, Mattson MP, Morris CM, Yao PJ (2008). Evidence for CALM in directing VAMP2 trafficking. Traffic.

[78] Kanatsu K, Morohashi Y, Suzuki M, Kuroda H, Watanabe T, Tomita T (2014). Decreased CALM expression reduces Aβ42 to total Aβ ratio through clathrin-mediated endocytosis of γ-secretase. Nat Commun.

[79] Xiao Q, Gil S-C, Yan P, Wang Y, Han S, Gonzales E (2012). Role of phosphatidylinositol clathrin assembly lymphoid-myeloid leukemia (PICALM) in intracellular amyloid precursor protein (APP) processing and amyloid plaque pathogenesis. JBC.

[80] Treusch S, Hamamichi S, Goodman JL, Matlack KES, Chung CY, Baru V (2011). Functional links between Aβ toxicity, endocytic trafficking, and Alzheimer’s disease risk factors in yeast. Science.

[81] Tian Y, Chang JC, Fan EY, Flajolet M, Greengard P (2013). Adaptor complex AP2/PICALM, through interaction with LC3, targets Alzheimer’s APP-CTF for terminal degradation via autophagy. PNAS U S A.

[82] Johnson ECB, Dammer EB, Duong DM, Yin L, Thambisetty M, Troncoso JC (2018). Deep proteomic network analysis of Alzheimer’s disease brain reveals alterations in RNA binding proteins and RNA splicing associated with disease. Mol Neurodegener.

[83] Raj Towfique, Li Yang I., Wong Garrett, Humphrey Jack, Wang Minghui, Ramdhani Satesh, Wang Ying-Chih, Ng Bernard, Gupta Ishaan, Haroutunian Vahram, Schadt Eric E., Young-Pearse Tracy, Mostafavi Sara, Zhang Bin, Sklar Pamela, Bennett David A., De Jager Philip L. (2018). Integrative transcriptome analyses of the aging brain implicate altered splicing in Alzheimer’s disease susceptibility. Nature Genetics.

[84] Osisami M, Ali W, Frohman MA (2012). A role for phospholipase D3 in myotube formation. PLoS One.

[85] Pedersen KM, Finsen B, Celis JE, Jensen NA (1998). Expression of a novel murine phospholipase D homolog coincides with late neuronal development in the forebrain. JBC.

[86] Satoh J-I, Kino Y, Yamamoto Y, Kawana N, Ishida T, Saito Y (2014). PLD3 is accumulated on neuritic plaques in Alzheimer’s disease brains. Alzheimers Res Ther.

[87] Mukadam AS, Breusegem SY, Seaman MNJ (2018). Analysis of novel endosome-to-Golgi retrieval genes reveals a role for PLD3 in regulating endosomal protein sorting and amyloid precursor protein processing. CMLS.

[88] Munck A, Böhm C, Seibel NM, Hashemol Hosseini Z, Hampe W (2005). Hu-K4 is a ubiquitously expressed type 2 transmembrane protein associated with the endoplasmic reticulum. FEBS J.

[89] Gonzalez AC, Schweizer M, Jagdmann S, Bernreuther C, Reinheckel T, Saftig P (2018). Unconventional trafficking of mammalian phospholipase D3 to lysosomes. Cell Rep.

[90] Breusegem SY, Seaman MNJ (2014). Genome-wide RNAi screen reveals a role for multipass membrane proteins in endosome-to-Golgi retrieval. Cell Rep.

[91] Fazzari P, Horre K, Arranz AM, Frigerio CS, Saito T, Saido TC (2017). PLD3 gene and processing of APP. Nature.

[92] Chakraborty A, de Wit NM, van der Flier WM, de Vries HE (2017). The blood brain barrier in Alzheimer’s disease. Vasc Pharmacol.

[93] Wang J, Gu BJ, Masters CL, Wang Y-J (2017). A systemic view of Alzheimer disease — insights from amyloid-β metabolism beyond the brain. Nat Rev Neurol.

[94] Juul Rasmussen Ida, Tybjærg-Hansen Anne, Rasmussen Katrine Laura, Nordestgaard Børge G., Frikke-Schmidt Ruth (2019). Blood–brain barrier transcytosis genes, risk of dementia and stroke: a prospective cohort study of 74,754 individuals. European Journal of Epidemiology.

[95] Storck SE, Hartz AMS, Bernard J, Wolf A, Kachlmeier A, Mahringer A (2018). The concerted amyloid-beta clearance of LRP1 and ABCB1/P-gp across the blood-brain barrier is linked by PICALM. Brain Behav Immun.

[96] Parikh I, Fardo DW, Estus S (2014). Genetics of PICALM expression and Alzheimer’s disease. PLoS One.

[97] Yates PA, Desmond PM, Phal PM, Steward C, Szoeke C, Salvado O (2014). Incidence of cerebral microbleeds in preclinical Alzheimer disease. Neurology.

[98] Iturria-Medina Y, Sotero RC, Toussaint PJ, Mateos-Pérez JM, Evans AC, Alzheimer’s Disease Neuroimaging Initiative MW (2016). Early role of vascular dysregulation on late-onset Alzheimer’s disease based on multifactorial data-driven analysis. Nat Commun.

[99] Snowdon DA, Greiner LH, Mortimer JA, Riley KP, Greiner PA, Markesbery WR (1997). Brain infarction and the clinical expression of Alzheimer disease. The Nun study. JAMA.

[100] Cochran JN, Rush T, Buckingham SC, Roberson ED (2015). The Alzheimer’s disease risk factor CD2AP maintains blood-brain barrier integrity. Hum Mol Genet.

[101] Li C, Ruotsalainen V, Tryggvason K, Shaw AS, Miner JH (2000). CD2AP is expressed with nephrin in developing podocytes and is found widely in mature kidney and elsewhere. Am J Physiol Renal Physiol.

[102] Tsuji K, Păunescu TG, Suleiman H, Xie D, Mamuya FA, Miner JH (2017). Re-characterization of the Glomerulopathy in CD2AP deficient mice by high-resolution helium ion scanning microscopy. Sci Rep.

[103] van Duijn TJ, Anthony EC, Hensbergen PJ, Deelder AM, Hordijk PL (2010). Rac1 recruits the adapter protein CMS/CD2AP to cell-cell contacts. JBC.

[104] Schaefer A, van Duijn TJ, Majolee J, Burridge K, Hordijk PL (2017). Endothelial CD2AP binds the receptor ICAM-1 to control Mechanosignaling, leukocyte adhesion, and the route of leukocyte diapedesis in vitro. J Immunol.

[105] Thomas Sunil, Hoxha Kevther, Alexander Walker, Gilligan John, Dilbarova Rima, Whittaker Kelly, Kossenkov Andrew, Prendergast George C., Mullin James M. (2018). Intestinal barrier tightening by a cell-penetrating antibody to Bin1, a candidate target for immunotherapy of ulcerative colitis. Journal of Cellular Biochemistry.

[106] Mäger I, Meyer AH, Li J, Lenter M, Hildebrandt T, Leparc G (2017). Targeting blood-brain-barrier transcytosis – perspectives for drug delivery. Neuropharmacology.

[107] Schwartz CM, Cheng A, Mughal MR, Mattson MP, Yao PJ (2010). Clathrin assembly proteins AP180 and CALM in the embryonic rat brain. J Comp Neurol.

[108] Waldau B, Shetty AK (2008). Behavior of neural stem cells in the Alzheimer brain. CMLS.

[109] Moreno-Jiménez EP, Flor-García M, Terreros-Roncal J, Rábano A, Cafini F, Pallas-Bazarra N (2019). Adult hippocampal neurogenesis is abundant in neurologically healthy subjects and drops sharply in patients with Alzheimer’s disease. Nat Med.

[110] Cummins TD, Wu KZL, Bozatzi P, Dingwell KS, Macartney TJ, Wood NT (2018). PAWS1 controls cytoskeletal dynamics and cell migration through association with the SH3 adaptor CD2AP. J Cell Sci.

[111] Monzo P, Gauthier NC, Keslair F, Loubat A, Field CM, Le Marchand-Brustel Y (2005). Clues to CD2-associated protein involvement in cytokinesis. Mol Biol Cell.

[112] Schiffer M, Mundel P, Shaw AS, Böttinger EP (2004). A novel role for the adaptor molecule CD2-associated protein in transforming growth factor-beta-induced apoptosis. JBC.

[113] Desrochers G, Cappadocia L, Lussier-Price M, Ton A-T, Ayoubi R, Serohijos A (2017). Molecular basis of interactions between SH3 domain-containing proteins and the proline-rich region of the ubiquitin ligase Itch. JBC.

[114] Rossi M, Aqeilan RI, Neale M, Candi E, Salomoni P, Knight RA (2006). The E3 ubiquitin ligase Itch controls the protein stability of p63. PNAS.

[115] Althubiti M, Lezina L, Carrera S, Jukes-Jones R, Giblett SM, Antonov A (2014). Characterization of novel markers of senescence and their prognostic potential in cancer. Cell Death Dis.

[116] Nagaoka-Yasuda R, Matsuo N, Perkins B, Limbaeck-Stokin K, Mayford M (2007). An RNAi-based genetic screen for oxidative stress resistance reveals retinol saturase as a mediator of stress resistance. Free Radic Biol Med.

[117] Suzuki M, Tanaka H, Tanimura A, Tanabe K, Oe N, Rai S (2012). The clathrin assembly protein PICALM is required for erythroid maturation and transferrin internalization in mice. PLoS One.

[118] Scotland PB, Heath JL, Conway AE, Porter NB, Armstrong MB, Walker JA (2012). The PICALM protein plays a key role in iron homeostasis and cell proliferation. PLoS One.

[119] Bartzokis G, Lu PH, Mintz J (2007). Human brain myelination and amyloid beta deposition in Alzheimer’s disease. Alzheimers Dement.

[120] Adams SL, Tilton K, Kozubek JA, Seshadri S, Delalle I (2016). Subcellular changes in bridging integrator 1 protein expression in the cerebral cortex during the progression of Alzheimer disease pathology. J Neuropathol Exp Neurol.

[121] Cabrera-Serrano Macarena, Mavillard Fabiola, Biancalana Valerie, Rivas Eloy, Morar Bharti, Hernández-Laín Aurelio, Olive Montse, Muelas Nuria, Khan Eduardo, Carvajal Alejandra, Quiroga Pablo, Diaz-Manera Jordi, Davis Mark, Ávila Rainiero, Domínguez Cristina, Romero Norma Beatriz, Vílchez Juan J., Comas David, Laing Nigel G., Laporte Jocelyn, Kalaydjieva Luba, Paradas Carmen (2018). A Roma founderBIN1mutation causes a novel phenotype of centronuclear myopathy with rigid spine. Neurology.

[122] Braak H, Braak E (1996). Development of Alzheimer-related neurofibrillary changes in the neocortex inversely recapitulates cortical myelogenesis. Acta Neuropathol.

[123] McKenzie AT, Moyon S, Wang M, Katsyv I, Song W-M, Zhou X (2017). Multiscale network modeling of oligodendrocytes reveals molecular components of myelin dysregulation in Alzheimer’s disease. Mol Neurodegener.

[124] Pak K, Chan SL, Mattson MP (2003). Presenilin-1 mutation sensitizes oligodendrocytes to glutamate and amyloid toxicities, and exacerbates white matter damage and memory impairment in mice. NeuroMolecular Med.

[125] Bergles DE, Roberts JD, Somogyi P, Jahr CE (2000). Glutamatergic synapses on oligodendrocyte precursor cells in the hippocampus. Nature.

[126] Pericak-Vance MA, Bebout JL, Gaskell PC, Yamaoka LH, Hung WY, Alberts MJ (1991). Linkage studies in familial Alzheimer disease: evidence for chromosome 19 linkage. Am J Hum Genet.

[127] Michaelson DM (2014). APOE ε4: the most prevalent yet understudied risk factor for Alzheimer’s disease. Alzheimers Dement.

[128] Shi Z, Yu H, Wu Y, Ford M, Perschon C, Wang C (2018). Genetic risk score modifies the effect of *APOE* on risk and age onset of Alzheimer’s disease. Clin Genet.

[129] Sleegers K, Bettens K, De Roeck A, Van Cauwenberghe C, Cuyvers E, Verheijen J (2015). A 22-single nucleotide polymorphism Alzheimer’s disease risk score correlates with family history, onset age, and cerebrospinal fluid Aβ42. Alzheimers Dement.

[130] Huang Y-WA, Zhou B, Wernig M, Südhof TC (2017). ApoE2, ApoE3, and ApoE4 differentially stimulate APP transcription and Aβ secretion. Cell.

[131] Kuszczyk MA, Sanchez S, Pankiewicz J, Kim J, Duszczyk M, Guridi M (2013). Blocking the interaction between apolipoprotein E and Aβ reduces Intraneuronal accumulation of Aβ and inhibits synaptic degeneration. Am J Pathol.

[132] Liu C-C, Zhao N, Fu Y, Wang N, Linares C, Tsai C-W (2017). ApoE4 accelerates early seeding of amyloid pathology. Neuron.

[133] Prasad H, Rao R (2018). Amyloid clearance defect in ApoE4 astrocytes is reversed by epigenetic correction of endosomal pH. PNAS.

[134] Chen Y, Durakoglugil MS, Xian X, Herz J (2010). ApoE4 reduces glutamate receptor function and synaptic plasticity by selectively impairing ApoE receptor recycling. PNAS U S A..

[135] Zhao N, Liu C-C, Van Ingelgom AJ, Martens YA, Linares C, Knight JA (2017). Apolipoprotein E4 impairs neuronal insulin signaling by trapping insulin receptor in the endosomes. Neuron.

[136] Nuriel T, Peng KY, Ashok A, Dillman AA, Figueroa HY, Apuzzo J (2017). The endosomal-lysosomal pathway is dysregulated by APOE4 expression in vivo. Front Neurosci.

[137] Zhu L, Zhong M, Elder GA, Sano M, Holtzman DM, Gandy S (2015). Phospholipid dysregulation contributes to ApoE4-associated cognitive deficits in Alzheimer’s disease pathogenesis. PNAS U S A.

[138] George AA, Hayden S, Stanton GR, Brockerhoff SE (2016). Arf6 and the 5’phosphatase of synaptojanin 1 regulate autophagy in cone photoreceptors. Insid Cell.

[139] Garai K, Baban B, Frieden C (2011). Self-association and stability of the ApoE isoforms at low pH: implications for ApoE-lipid interactions. Biochemistry.

[140] Morrow JA, Hatters DM, Lu B, Höchtl P, Oberg KA, Rupp B (2002). Apolipoprotein E4 forms a molten globule. JBC.

[141] Dafnis I, Argyri L, Sagnou M, Tzinia A, Tsilibary EC, Stratikos E (2016). The ability of apolipoprotein E fragments to promote intraneuronal accumulation of amyloid beta peptide 42 is both isoform and size-specific. Sci Rep.

[142] Mazur-Kolecka B, Kowal D, Sukontasup T, Dickson D, Frackowiak J (2003). The effect of oxidative stress on accumulation of apolipoprotein E3 and E4 in a cell culture model of beta-amyloid angiopathy (CAA). Brain Res.

[143] Ma Q, Zhao Z, Sagare AP, Wu Y, Wang M, Owens NC (2018). Blood-brain barrier-associated pericytes internalize and clear aggregated amyloid-β42 by LRP1-dependent apolipoprotein E isoform-specific mechanism. Mol Neurodegener.

[144] Shinohara M, Koga S, Konno T, Nix J, Shinohara M, Aoki N (2017). Distinct spatiotemporal accumulation of N-truncated and full-length amyloid-β42 in Alzheimer’s disease. Brain.

[145] Jonsson T, Stefansson H, Steinberg S, Jonsdottir I, Jonsson PV, Snaedal J (2013). Variant of *TREM2* associated with the risk of Alzheimer’s disease. N Engl J Med.

[146] Song W, Hooli B, Mullin K, Jin SC, Cella M, Ulland TK (2017). Alzheimer’s disease-associated TREM2 variants exhibit either decreased or increased ligand-dependent activation. Alzheimers Dement.

[147] Schlepckow K, Kleinberger G, Fukumori A, Feederle R, Lichtenthaler SF, Steiner H (2017). An Alzheimer-associated TREM2 variant occurs at the ADAM cleavage site and affects shedding and phagocytic function. EMBO Mol Med.

[148] Lessard CB, Malnik SL, Zhou Y, Ladd TB, Cruz PE, Ran Y (2018). High-affinity interactions and signal transduction between Aβ oligomers and TREM2. EMBO Mol Med.

[149] Claes Christel, Van Den Daele Johanna, Boon Ruben, Schouteden Sarah, Colombo Alessio, Monasor Laura Sebastian, Fiers Mark, Ordovás Laura, Nami FatemehArefeh, Bohrmann Bernd, Tahirovic Sabina, De Strooper Bart, Verfaillie Catherine M. (2019). Human stem cell–derived monocytes and microglia-like cells reveal impaired amyloid plaque clearance upon heterozygous or homozygous loss of TREM2. Alzheimer's & Dementia.

[150] Raha-Chowdhury R, Henderson JW, Raha AA, Stott SRW, Vuono R, Foscarin S (2018). Erythromyeloid-derived TREM2: a major determinant of Alzheimer’s disease pathology in Down syndrome. J Alzheimers Dis.

[151] Atagi Y, Liu C-C, Painter MM, Chen X-F, Verbeeck C, Zheng H (2015). Apolipoprotein E is a ligand for triggering receptor expressed on myeloid cells 2 (TREM2). JBC.

[152] Krasemann S, Madore C, Cialic R, Baufeld C, Calcagno N, El Fatimy R (2017). The TREM2-APOE pathway drives the transcriptional phenotype of dysfunctional microglia in neurodegenerative diseases. Immunity.

[153] Ulland TK, Song WM, Huang SC-C, Ulrich JD, Sergushichev A, Beatty WL (2017). TREM2 maintains microglial metabolic fitness in Alzheimer’s disease. Cell.

[154] Lucin KM, O’Brien CE, Bieri G, Czirr E, Mosher KI, Abbey RJ (2013). Microglial Beclin 1 regulates Retromer trafficking and phagocytosis and is impaired in Alzheimer’s disease. Neuron.

[155] Huang K-L, Marcora E, Pimenova AA, Di Narzo AF, Kapoor M, Jin SC (2017). A common haplotype lowers PU.1 expression in myeloid cells and delays onset of Alzheimer’s disease. Nat Neurosci.

[156] Sweeney MD, Sagare AP, Zlokovic BV (2018). Blood–brain barrier breakdown in Alzheimer disease and other neurodegenerative disorders. Nat Rev Neurol.

[157] Bien-Ly N, Yu YJ, Bumbaca D, Elstrott J, Boswell CA, Zhang Y (2014). Transferrin receptor (TfR) trafficking determines brain uptake of TfR antibody affinity variants. J Exp Med.

[158] Sonvico F, Clementino A, Buttini F, Colombo G, Pescina S, Stanisçuaski Guterres S (2018). Surface-modified Nanocarriers for nose-to-brain delivery: from bioadhesion to targeting. Pharmaceutics.

[159] Espuny-Camacho I, Arranz AM, Fiers M, Snellinx A, Ando K, Munck S (2017). Hallmarks of Alzheimer’s disease in stem-cell-derived human neurons transplanted into mouse brain. Neuron.

[160] Tharkeshwar Arun Kumar, Gevaert Kris, Annaert Wim (2018). Front Cover: Organellar Omics - A Reviving Strategy to Untangle the Biomolecular Complexity of the Cell. PROTEOMICS.

[161] Thimiri Govinda Raj DB, Ghesquiere B, Tharkeshwar AK, Coen K, Derua R, Vanderschaeghe D (2014). A novel strategy for the comprehensive analysis of the biomolecular composition of isolated plasma membranes. Mol Syst Biol.

[162] Tharkeshwar AK, Trekker J, Vermeire W, Pauwels J, Sannerud R, Priestman DA (2017). A novel approach to analyze lysosomal dysfunctions through subcellular proteomics and lipidomics: the case of NPC1 deficiency. Sci Rep.

[163] Abu-Remaileh M, Wyant GA, Kim C, Laqtom NN, Abbasi M, Chan SH (2017). Lysosomal metabolomics reveals V-ATPase- and mTOR-dependent regulation of amino acid efflux from lysosomes. Science (80- ).

[164] van Blitterswijk M, van Es MA, Hennekam EAM, Dooijes D, van Rheenen W, Medic J (2012). Evidence for an oligogenic basis of amyotrophic lateral sclerosis. Hum Mol Genet.

[165] Morgan S, Shatunov A, Sproviero W, Jones AR, Shoai M, Hughes D (2017). A comprehensive analysis of rare genetic variation in amyotrophic lateral sclerosis in the UK. Brain.

[166] Lesage S, Drouet V, Majounie E, Deramecourt V, Jacoupy M, Nicolas A (2016). Loss of VPS13C function in autosomal-recessive parkinsonism causes mitochondrial dysfunction and increases PINK1/Parkin-dependent mitophagy. Am J Hum Genet.

[167] Bonvicini C, Scassellati C, Benussi L, Di Maria E, Maj C, Ciani M (2019). Next generation sequencing analysis in early onset dementia patients. J Alzheimers Dis.

