# Supplementary material for: Endo-lysosomal dysregulations and late-onset Alzheimer’s disease: impact of genetic risk factors
Source: Mol Neurodegener. 2019 Jun 3;14:20. doi: 10.1186/s13024-019-0323-7 (PMC6547588; doi:10.1186/s13024-019-0323-7)
Supplement: Supplementary file 1 — Table S1. Overview of studies on BIN1, CD2AP, PICALM and PLD3 expression levels. The table summarizes the transcript and protein levels of the LOAD risk genes, as detected in in vitro and in vivo studies, as well as the subcellular localization of the associated protein when studied. N.d.; not determined. Table S.2 LOAD-associated SNPs. Overview of the different SNPs within BIN1, CD2AP, PICALM and PLD3 that are linked to LOAD development. If studied, the associated clinical effects are listed. Table S3. Summary of the known protein interactors of BIN1, CD2AP, PICALM and PLD3. The table further includes the model(s) in which the interaction was studied, as well as the validation method(s), involved region within the proteins, and observed effects downstream of binding. (DOCX 191 kb) [file 13024_2019_323_MOESM1_ESM.docx]

**Supplementary tables**

Table S1. LOAD-linked protein expressions and localizations

|  | **Model(s)** | **Transcript level** | **Protein level** | **Subcellular localization** | **Function link – remarks** | **Ref.** |
| --- | --- | --- | --- | --- | --- | --- |
| **BIN1 levels** | In vitro; HeLa cells | n.d. | n.d. | Endosomal structures; ER, early to late endosomes and lysosomes | n.d. | [1] |
|  | In vitro; HeLa cells, EpH4 cells | n.d. | n.d. | Endocytic recycling compartment | n.d. | [2] |
|  | In vivo; rat brain | n.d. | + Neurons  - Glia | Only full-length neuronal variant detected at synaptic terminals | n.d. | [3] |
|  | In vivo; human brain | + Brain | + Brain | Axon initial segments and nodes of Ranvier | Discrepant mRNA-protein level; technical origin, translational and/or high protein turnover | [4] |
|  | In vivo; human brain | n.d. | + Brain | Both cytosolic and nuclear, though not uniformly present | Inverse relationship: BIN1 and cell proliferation | [5] |
|  | In vivo; human non-AD and AD brains | n.d. | * Health: ++ oligodendrocytes, neurons, + astrocytes  * AD: downregulated in neurons | * Health: axonal localization  * AD: switch to the cytoplasm | Neuritic plaques co-localize with loss of BIN1 neuropil staining | [6] |
|  | In vivo; human non-AD and AD brains | + Parietal lobes | n.d. | n.d. | Higher levels linked to later age at onset & faster disease course | [7] |
|  | In vivo; human non-AD and AD brains | ° Health: ++ oligodendroglia, + neurons, - other cells  ° AD: 18% more mRNA with ex7 | ° Health: white matter levels ~4-fold higher than grey matter’s  ° AD: lowered neuronal BIN1 and increased LMW oligodendroglia isoforms of BIN1 | Along axons of perforant path and alveus, no specific localization at nodes or at paranodal area | ~90% of transcripts in white matter lack ex7; oligo lower MW ~65-75 kDa BIN1 isoforms | [8] |
|  | In vivo; human non-AD and AD brains | AD: increased in frontal cortices | ++ neurons  + microglia  - astrocytes | Co-localization with tau; follows axonal neurofilament labellin | Modulator of tau pathology | [9] |
|  | In vivo; human non-AD and AD brains | n.d. | + Oligodendroglia & processes in neuropil (+) and white matter (++)  - Microglia and astrocytes | Weak cytoplasmic signal in neurons | ° Absent in regions with Aβ deposits  ° BIN1 Hypothesized to not take part in AD inflammatory response | [10] |
| **CD2AP levels** | In vitro; HeLa cells | n.d. | n.d. | ° Punctate cytoplasmic structures  ° Transiently in membrane ruffles  ° Together with EGFR after stimulus | Regulates trafficking of internalized EGFR | [11] |
|  | In vitro; CHO cells | n.d. | n.d. | ° Cytoplasmic vesicular structures  ° Aligned along actin fibers or ruffles | CD2AP dimerization is necessary for membrane localization | [12] |
|  | In vitro; mouse cortical neurons | n.d. | n.d. | Co-localization with APP vesicles and Rab5 twice as high in dendrites as in axons (polarized association) | Role in APP transport to lysosomes for degradation | [13] |
|  | In vitro; mouse podocytes | n.d. | n.d. | ° ER- & Golgi-enriched fraction  ° Insulin-dependent dispersion from perinuclear region into cytoplasm | n.d. | [14] |
|  | In vivo; rat dorsal root ganglion neurons and brains | Up- (ganglion) and down- (brain) regulated along postnatal development | Adult: + cerebellum, cerebral cortex; primarily localized in blood vessel epithelia and axonal projections | ° Punctate in neurites and soma, and accretes at F-actin^+^/tubulin^−^ neurite tips, branch points and swellings  ° Colocalization with Rab5 | ° Transcription is plasticity context-dependent; NGF induces CD2AP 1.90-fold  ° Link with adult axonal sprouting | [15] |
|  | In vivo; mouse neurvous system | n.d. | ° Embryonic: E11-E17 paraspinal ganglia, E15-E17 choroid plexus  ° Adult: + brain | n.d. | *CD2AP* expression is spatio-temporal regulated; possible role in tissue/cell differentiation | [16] |
|  | In vivo; mouse brain | n.d. | Strong staining in capillaries | n.d. | n.d. | [17] |
| **PICALM levels** | In vitro; HeLa cells | n.d. | n.d. | Shallow and deep clathrin-coated pits or vesicles | n.d. | [18] |
|  | In vitro; HeLa cells | n.d. | n.d. | ° On autophagic precursors  ° Co-localization with ATG16L1 | Role in the initial steps of autophagosome formation | [19] |
|  | In vitro; HeLa cells and COS-1 cells | n.d. | ° 66 kDa variant: +++ COS-1, ++ HeLa  ° 72 kDa variant: ++ HeLa, + COS-1 | ° Plasma membrane–coated pits  ° Golgi apparatus | Component of coated pit internalization machinery | [20] |
|  | In vitro; neuroglioma H4 cells, expressing endogenous APP | n.d. | Two isofroms detected  ° 72.2 kDa band (predicted 70.6 kDa)  ° 65.8 kDa band (predicted 66.3 kDa) | n.d. | KD of either of the variants on itself has no effect on cleavage of APP | [21] |
|  | In vitro; human frontal cortex AD endothelial cells | Downregulated by ~34% as compared to controls | Downregulated by ~35% as compared to controls | n.d. | ~50% reduced basolateral–to–apical transcytosis of Aβ | [22] |
|  | In vitro; endothelium of rs3851179 A allele iPSC | ~78% higher | ~72% higher | n.d. | 120% higher Aβ clearance | [22] |
|  | In vivo; rat CA1 HC at P10 and P37 | n.d. | n.d. | ~40% in intracellular regions; seldom CCV of TGN, often early/ sorting endosomes + tubulovesicular structures near dendrite branch point | Interaction with CCV is expected to be transient | [23] |
|  | In vivo; rats | n.d. | Developing hippocampus: ++ E18, ++ P10, + P14, + P30 | CA1 stratum radiatum (P2/10): equal spreading across synaptic junction, also at tip dendritic ﬁlopodium, in endosomes and internal CCV | n.d. | [24] |
|  | In vivo; Sprague-Dawley rats | n.d. | ° + young (Tuj1^+^) and mature (NeuN^+^) postmitotic neurons, neural progenitors (Sox2^+^) and pluripotent ESC (SSEA4^+^/Nanog^+^)  ° Long variant increases along brain development, reverse relationship for short variant | n.d. | Function in neural development | [25] |
|  | In vivo; rats | ° Short variant: neurons  ° Long variant: derived from glia | ° Short variant: + hippocampus, + cerebellum  ° Long variant: ++ hippocampus, ++ cerebellum | ° Neurons: 37% both pre- and post-synaptic (if pre-synaptic focused in or in the vicinity of synaptic vesicles)  ° Surrounding astroglial processes | Accessory protein involved in synaptic exo-endocytic vesicle cycling | [26] |
|  | In vivo; hAPP^V717I^ carrying mouse cortices | n.d. | ° 3 variants of 72, 68 and 62 kDa  ° 72/68 bands unaffected by age (3-18 m), or AD pathology  ° 62 kDa level increases with age in controls, inverse trend in AD mice -> 6-fold change at 18 m | n.d. | n.d. | [27] |
|  | In vivo; APP/PS1 mouse Cortex & HC | n.d. | + neurons (NeuN^+^ ), - astrocytes (GFAP^+^) and microglia (Iba1^+^) | n.d. | n.d. | [28] |
|  | In vivo; AD, C9orf72 ALS, Pick disease, LBD, FTLD, PSP & CBD patients | n.d. | ++ endothelial cells-blood vessels  + neurons and microglia | ° NFTs of AD and PSP and Pick disease  ° Co-localization with P-tau in AD (3R + 4R), PSP (4R) and Pick disease (3R) | Inverse relationship with levels of autophagic markers LC3-II and Beclin-1; possible PICALM depletion-induced autophagic dysfunction | [29] |
|  | In vivo; human non-AD and FAD^APP^ brains | n.d. | ° ~25 % decrease of full length  ° 132% and 428% the baseline of cleaved 25 and 50 kDa species  ° Health: + neuronal perinuclear rim, ++ endothelial cells & epithelial lining of choroid plexus  ° AD: Unaltered endo/epithelial stain, increased neuronal level | n.d. | PICALM present in over 85 % of NFTs, but not in pre-tangles or in extracellular ghost tangles | [30] |
|  | In vivo; human non-AD and LOAD brains | n.d. | ° ~40 % decrease of full length  ° 141% and 983% the baseline of cleaved 25 and 50 kDa species  ° LOAD: elevated in HC & cortex microglia, slightly in astrocytes, not in oligodendroglia | n.d. | Calpain and caspase-3 generate shorter PICALM variants, with calpain’s efficiency being higher for generating 50 kDa | [30] |
|  | In vivo; human non-AD and AD brains | n.d. | ° Health: 65% of endothelia  ° Braak stage V–VI: decreased in endothelia (by 65%), but slightly increased in neurons | n.d. | Aβ is not a PICALM inhibitor; plaques in endothelial cultures unalter PICALM levels | [22] |
|  | In vivo; human non-AD and AD brains | ° Linear regression: microvessels (p = 4.0×10^−3^), neurons (p = 4.2×10^−6^), astroglia (1.8×10^−5^) | Anterior cingulate tissue: ++ microvessels, + neurons, + glia | n.d. | Hypothesized to help with Aβ clearance across the blood–brain barrier | [31] |
|  | In vivo; Human non-AD and AD brains | ° Increase in AD-frontal cortices, but not the temporal cortex or thalamus | ° ++ micro-vessel walls, + neurons, + glia  ° No localization in Aβ-plaques or tangles | n.d. | Region specificity: could P-Tau modulate Aβ impact on PICALM expression? | [32] |
| **PLD3 levels** | In sillico; ommercial human multiple tissue arrays | ° 2200 bp: ubiquitously, lowest in leukocytes  ° 1700 bp: +++ brain, spleen, ++ heart, skeletal muscle, lung, + rest | n.d. | Glycosylation-dependent, type 2 transmembrane protein | Variances in 5′‐UTR sequences impact transcription | [33] |
|  | In vitro; HeLa and SH-SY5Y cells | n.d. | n.d. | ° Co-localizes with APP (40-80%)  ° Present in (post)-Golgi membranes,  Vps35^+^ endosomal membranes | Functioning as an endosome-to-Golgi retrieval protein | [34] |
|  | In vitro; HeLa and SH-SY5Y cells | n.d. | n.d. | ° Luminal domain: mainly lysosomal  ° Full-length protein and 12-kDa membrane-bound N-terminal fragment: endosomes, Golgi and ER | Undergoes proteolysis; acidic pH and cysteine proteases  ° Is ESCRT- and ubiquitin-dependently transported to lysosomes | [35] |
|  | In vitro; HCT116 human colorectal cancer cell line | n.d. | Levels are halved under 1% oxygen levels (hypoxic stress) | n.d. | Regulation HIF1-independent and O_2_-tension dependent (p<0.0001) | [36] |
|  | In vitro; C2C12 mouse myoblast cell line | Increased along differentiation, with highest levels reached at 4 post-induction | Differentiation-linked upsurge further increased by ER-stress | Relocalizes during myotube formation from ER subdomains to mitochondrial tubules | Possibly involved in lipid transfer/synthesis when tubules of ER wrap around those of mitochondria, inducing fission | [37] |
|  | In vitro; NIH/3T3 mouse fibroblast cell line | n.d. | n.d. | Anchored to ER membrane through transmembrane region AA_37–60_ | n.d. | [37] |
|  | In vivo; mouse tissue | n.d. | ++ cortical and HC pyramidal neurons, - HC interneurons and glial cells of the corpus callosum | Localization in LAMP1^+^ compartments and not in early endosomes | n.d. | [38] |
|  | In vivo; mouse tissue | ° ++ brains (especially forebrain), no to weak signal in non-nervous tissues  ° Neuronal origin, no to weak expression in neuroglia and Schwann cells | n.d. | n.d. | Expression elevated upon reaching late stages of neurogenesis | [39] |
|  | In vivo; mouse bone marrow hematopoietic stem cells | About 10-fold higher levels in cells that maintain their replication potential | n.d. | n.d. | Role in regulating the durable hematopoietic stem cell self-renewal potential | [40] |
|  | In vivo; human tissue | High across the brain, only low in corpus striatum, occipital lobe and visual cortex | Wide distribution, i.e across brain, cerebellum, liver, kidney, lung and heart | n.d. | Contributes to general/non-cell type-specific mechanisms | [41] |
|  | In vivo; human brain of cognitive normal individuals | ++ frontal, temporal, and occipital cortices and hippocampus | n.d. | n.d. | High transcription rates in regions that are vulnerable to AD pathology | [42] |
|  | In vivo; human non-AD and AD brains | Expression is about halved in AD brains | ° In neurons and a subset of pericytes, not in astrocytes, oligodendrocytes and microglia  ° Levels are about halved in AD brains | ° Soma and proximal neurites of cortical and HC-pyramidal neurons, and dentate gyrus granule cells  ° Senile plaques, only in AD cases | Accumulation in senile plaques is generally associated with the depletion of cellular levels | [43] |
|  | In vivo; AD patients | ° Significantly lower relative to healthy level  ° Inverse correlation with those of APP | n.d. | n.d. | Variant A442A leads to lower total PLD3 transcript levels | [42] |
| AD; Alzheimer's disease, CBD; corticobasal degeneration, CCV; cathrin-coated vesicles,HC, ESC; embryonic stem cell, FTLD; frontotemporal lobar degeneration, HC; hippocampus, LOAD; Late-onset Alzheimer disease, NFT, neurofibrillary tangle, PSP; progressive supranuclear palsy, TGN; trans-Golgi network, vWF; Von Willebrand factor. | | | | | | |

Table S2. LOAD-associated SNPs

| **Gene** | **SNP** | **Alleles (F-strand)** | **Risk allele** | **Population** | **Effect** | **Ref.** |
| --- | --- | --- | --- | --- | --- | --- |
| ***Bin1*** | rs744373 | G/A | G | Caucasians of CHARGE, TGEN, Mayo AD GWAS and GERAD1 database | Genome-wide significant association with LOAD: OR of 1.13 per minor allele copy; p=1.6×10^−11^ | [44] |
|  |  |  |  | Asian and Caucasian, meta-analysis of 54 articles | ° Statistically significant risk factor of AD onset  ° Higher AD risk in Caucasians: OR of 1.18 | [45] |
|  |  |  |  | Young healthy Chinese university students | GG carriers: lower working memory performance and hippocampal functional connectivity | [46] |
|  |  |  |  | MCI patients according Petersen  criteria for amnestic MCI from Spain | No significant association with the risk of conversion from MCI to AD or with disease progression | [47] |
|  | rs6733839 | C/T | T | Caucasians of ADNI cohort | Linked to volume decrease of right inferior parietal lobule, an association area flexible reconfiguring one’s behaviour | [48] |
|  |  |  |  | ADNI cohort | Associated with increased AD-linked brain shape asymmetry | [49] |
|  |  |  |  | Israeli Jewish (≥65 years old) with Type 2 Diabetes | Association with performance in episodic memory | [50] |
|  | rs7561528 | A/G | A | LOAD cases from NACC, NCRAD, NIA-funded AD Centres | LOAD susceptibility: p = 4.0E-14 - 5.2E-14 | [51] |
|  |  |  |  | LOAD patients with family history of AD from NIA-LOAD Family Study & NCRAD | Significant LOAD risk: p = 0.009 with, and p = 0.03 without, APOE adjustment | [52] |
|  |  |  |  | ADNI database | Association with entorhinal cortical and temporal pole cortical thickness | [53] |
|  |  |  |  | Autopsy cohort of cognitive healthy subjects of ADNI, MAP, ROS and Arizona *APOE* consortia | No link with neuritic plaque pathologic burden (p = 0.757) | [54] |
|  | rs12989701 | A/C | A | Caucasians of ADNI and GenADA cohort | ° Genome-wide association: p = 0.002, OR of 1.34  ° Low linkage disequilibrium with rs744373 (r^2^= 0.05)  ° Positioned in evolutionarily conserved region | [55] |
|  | rs59335482 | n.a. | Insertion of 3 C-bases | EADI1, GERAD1 and Belgium-Flanders case-control study samples | ° Linked to axonal neurofilament  ° Increases Tau load, no effect on Aβ40/42 levels  ° In linkage disequilibrium with rs744373 | [9] |
| ***CD2AP*** | rs9296559 | C/T | C | MCI patients according Petersen  criteria for amnestic MCI from Spain | ° C allele carriers progress more rapidly from mild cognitive impairment to AD (hazard ratio: 1.69)  ° No link with the risk of MCI to AD conversion in se | [47] |
|  | rs9349407 | C/G | C | LOAD cases from NACC, NCRAD, NIA-funded AD Centres | LOAD susceptibility: p = 8.6E-9 | [51] |
|  |  |  |  | GERAD1, EADI1, deCODE, AD-IG GWAS datasets & TGEN1, ADNI and Mayo1 datasets | Genome-wide association: p =  8.6E-9, OR of 1.11 | [56] |
|  |  |  |  | ADNI cohort | Impacts amyloid pathology: diminishes correlation between cortical PiB-binding and plasma apoE | [57] |
|  |  |  |  | Autopsy cohort of cognitive healthy subjects of ADNI, MAP, ROS and Arizona *APOE* consortia | Associated with increased neuritic plaque pathologic burden (p = 0.029) | [54] |
|  | rs10948363 | A/G | G | ADNI database | Promotes brain shape asymmetry irrespective of disease status | [49] |
| ***PICALM*** | rs561655 | A/G | G | Autopsy cohort of cognitive healthy subjects of ADNI, MAP, ROS and Arizona *APOE* consortia | No link with neuritic plaque pathologic burden (p = 0.117) | [54] |
|  |  |  |  | Meta-analysis; Caucasian, Arab, African American, and Caribbean Hispanic cases of ≥ 60 y (n = 7070) | ° OR of 0.89 in Caucasians, but not in other groups  ° Statistical interaction with APOE ε4 carrier status (p = 0.0024) | [58] |
|  | rs541458 | C/T | C | Caucasians with Italian ancestry | C carriers less detected in population of AD patients of 80+ years | [59] |
|  |  |  |  | Subjects of European descent | ° Associated with AD: p = 2.3E−11, OR of 0.87  ° TT carriers 20% decreased Aβ42 clearance through cerebrospinal fluid as compared to CC carriers | [60] |
|  | rs3851179 | T/C | C = risk  T = beneficial | Asian, Caucasian, and Yoruban | ° Genome-wide association: p =  1.9E-8, OR of 0.86  ° No interaction with *APOE* status, impacting AD risk | [61] |
|  |  |  |  | Meta-analysis; Caucasian, Arab, African American, and Caribbean Hispanic cases of ≥ 60 y (n = 7070) | ° OR of 0.89 in Caucasians, but not in other groups  ° Statistical interaction with APOE ε4 carrier status (p = 0.0068) | [58] |
|  |  |  |  | LOAD patients with family history of AD from NIA-LOAD Family Study & NCRAD | Confers risk primarily in APOE ε4^+^ subjects | [52] |
|  |  |  |  | Brazilian LOAD, AD, PD and control cases | Protective effect in ε4^-^ subjects, but absent in ε4^+^ carriers | [62] |
|  |  |  |  | MCI patients according Petersen  criteria for amnestic MCI from Spain | No significant association with the risk of conversion from MCI to AD or with disease progression | [47] |
|  |  |  |  | Danish (1905) birth cohort study, 92–93 years at intake | Male T-carriers present with improved cognitive composite scores, which is not seen in females | [63] |
|  |  |  |  | Individuals of SNAC-K study group | C risk allele reduces episodic memory performance | [64] |
|  |  |  |  | Elderly, Caucasian cohort from Mayo Clinic Rochester | C ‘risk’-linked allele shows slower advance to mild cognitive impairment/LOAD (hazard ratio of 0.85) | [65] |
|  |  |  |  | AD patients from German Dementia Competence Network | CC-carriers of ε4^+^ show reduced prefrontal cortex volumes and inferior episodic memory function | [66] |
|  |  |  |  | Subjects of the AddNeuroMed study and ADNI database | ° OD of 0.86  ° Linked to thicker entorhinal cortices | [67] |
|  |  |  |  | Samples from University of Kentucky AD Center Neuropathology Core | Increased total PICALM expression in anterior cingulate specimens | [31] |
|  |  |  |  | Inducible pluripotent stem cell-derived endothelium | ° TT carriers ~1,7-fold higher PICALM levels as CC  ° TT endocytes: 2-fold higher Aβ40 transcytosis | [22] |
|  |  |  |  | UK Brain Expression Consortium eQTL dataset, Trabzuni dataset | Risk allele decreases 65.8 kDa but not 72.2 kDa variant levels | [21] |
|  | rs10792832 | A/G | G | UK Brain Expression Consortium eQTL dataset, Trabzuni dataset | Risk allele decreases 65.8 kDa but not 72.2 kDa variant levels | [21] |
|  | rs17159904 | A/G | G | Individuals of Caribbean Hispanic ancestry | G allele just reaches a significant association with LOAD risk | [68] |
| ***PLD3*** | rs762406245 | T/G | G (M6R) | Cases from European and African-American descent | Associated to LOAD risk: p = 0.02, OR of 7.73 | [42] |
|  | rs145999145 | A/G | A (V232M) | Cases from European and African-American descent | ° V232M not detected in cognitively healthy elderlies ° Association to AD risk (OR of 2.10) and age of onset | [42] |
|  |  |  |  | WRAP participants, non-Hispanic Caucasian | ° Worsens story recall, visual learning and memory  ° Trend towards faster disease progression | [69] |
|  |  |  |  | Amsterdam Dementia cohort, Alzheimer Center Erasmus MC and RS | Nominally significant association with AD: p = 0.03, OR of 1.94 | [70] |
|  | rs4819 | G/A | A (A442A) | Cases from European and African-American descent | ° Associated to LOAD risk: p = 3.78E-7, OR of 2.12  ° Variant affects alternative splicing  ° Lowers full length mRNA levels and those with ex11 | [42] |
| German Alzheimer’s disease Integrated Genome Research Network (AD-IG), Alzheimer's Disease Neuroimaging Initiative (ADNI), Cohorts for Heart and Aging Research in Genomic Epidemiology (CHARGE), European Alzheimer Disease Initiative Investigators (EADI1), Genetic and Environmental Risk for Alzheimer's disease Consortium (GERAD1), Rush Memory and Aging Project (MAP), Mild Cognitive Impairment (MCI), National Alzheimer Coordinating Centre (NACC), National Cell Repository for Alzheimer's Disease (NCRAD), National Institute of Aging (NIA), odds ratio (OR), Religious Orders Study (ROS), Swedish National Study on Aging and Care in Kungsholmen (SNAC-K), Translational Genomics Research Institute (TGEN), Wisconsin Registry for Alzheimer's Prevention (WRAP). | | | | | | |

Table S3. LOAD-linked protein interactions

| **BIN1 interactors** | **Model(s)** | | **Identification** | **Validation** | **Region** | **Effect** | **Ref.** |
| --- | --- | --- | --- | --- | --- | --- | --- |
| Actin | | Recombinant proteins | Assay of co‐sedimentation | Electron microscopy, crosslinking assay | ° Binds F‐ and G‐actin  ° BIN1 BAR domain | ° Promotes actin polymerization  ° Stabilizes tau‐actin bundles | [71] |
| Beta-secretase 1 (BACE1) | | Recombinant proteins | GST pull-down assay – WB | n.d. | BIN1 BAR domain binds C’-intracellular BACE1 region | BACE1 domain is not post-translationally modified | [72] |
| Calmodulin | | Mouse brain lysates | Affinity chromatography – MS | n.d. | n.d. | Calcium hypothesis of AD | [73] |
| Charged multivesicular body protein 4B | | Mouse cardiomyocytes | Co-IP – WB | Super-resolution STORM imaging | BIN1 N-terminal BAR domain | Role in the ESCRT pathway to generate cell-cell communication microparticles | [74] |
| Clathrin | | Postnuclear supernatant fraction of rat brain | GST pull-down assay – WB | n.d. | BIN1 non-SH3 domain | Role in controlling vesicle endocytosis at synapses | [75] |
| Cytoplasmic linker protein CLIP-170 | | ° Human placental cDNA  ° HeLa cells | Y2H screen | Co-IP – WB | BIN1 interacts with internal cc-CLIP330-660 region | Formation of tubules that align with and are mechanistically-linked to microtubules | [76] |
| Dynamin | | Postnuclear supernatant fraction of rat brain | GST pull-down assay – WB | n.d. | BIN1 SH3 domain | Role in controlling vesicle endocytosis at synapses | [75] |
|  |  | Recombinant proteins | Electron microscopy | High-speed atomic force microscopy | n.d. | GTP-dependent membrane fission at 1:0.5-1 dynamin:BIN1 -> impacts vesicle size | [77] |
| E3 ubiquitin-protein ligase Itchy homolog (ITCH) | | Rat brain extracts | GST pull-down assay – MS | Mutation analysis, molecular modelling | Pro-rich region of ITCH (AA_255–264_) binds to BIN1 SH3 domain | Regulates immune response, hematopoietic lineage development and p15-linked apoptosis | [78] |
| EH domain-binding protein 1-like 1 (EHBP1L1) | | ° Mouse brain lysates  ° Caco-2 cell lysates | GST pull-down assay – MS | GST pull-down assay – WB | PR domain of EHBP1L1 binds to BIN1 C-terminal SH3 region | Control of epithelial apical-directed transport | [2] |
| Myc proto-oncogene protein | | ° Murine E10.5 cDNA  ° Recombinant proteins | Y2H screen | GST pull-down assay | N-terminal Myc box domains | Tumor suppressor; inhibits myc-induced malignant cell transformation | [79] |
| Ras and Rab Interactor 3 (RIN3) | | Human leukocyte cDNA library | Y2H screen | GST pull-down assay – WB | SH3 domain of BIN1 binds N-terminal region of RIN3 | As for CD2AP, the interaction entails localization at Rab5a^+^ early endosomes | [80] |
| Sorting nexin 4 (SNX4) | | ° Jurkat cell cDNA  ° 3T3 and HeLa cells | Y2H screen | Co-IP – WB | SNX4 N-terminus interacts with BIN1 AA147-304 | Role in endocytosis and intracellular trafficking | [1] |
| Synaptojanin | | Postnuclear supernatant fraction of rat brain | GST pull-down assay – WB | n.d. | BIN1 SH3 domain | Role in controlling vesicle endocytosis at synapses | [75] |
| Tau | | ° SH-SY5Y, HEK293 cells  ° Synaptosomes (mouse) | Co-localisation | Co-IP – WB, GST pull-down assay | n.d. | Modulator of AD-linked tau pathology | [9] |
|  |  | Recombinant proteins | NMR spectroscopy | Isothermal titration calorimetry | BIN1 SH3 domain binds to Tau Pro‐rich AA_210-240_ | Tau binding competes with an intramolecular interaction | [81] |
| **CD2AP interactors** |  | |  |  |  |  |  |
| Anillin | | ° Mouse cDNA library  ° HeLa cells | Y2H screening | GST pull-down assay – WB, co-localization | First two SH3 domains of CD2AP and N-terminal fragment (1-155) of anillin | Co-phosphorylated during early mitosis | [82] |
| Cluster of differentiation 2 | | ° Mouse thymus cDNA  ° HeLa and Jurkat T cells | Y2H screening | Co-IP – WB, surface plasmon resonance | First SH3 domain of CD2AP binds cytoplasmic tail of CD2 (Kd of 130 nM) | ° Co-expressed in T and NK cells  ° T cell activation promotes interaction  ° Role in T-cell receptor patterning | [83] |
| Cortactin | | MDA-MB-231 cell lysates | GST pull-down assay – MS | Co-IP – WB | Cortactin SH3 domain binds CD2AP Pro-rich sequence 2 | Role in growth factor receptor endocytosis and trafficking (EGFR) | [11] |
| Clathrin heavy chain | | Mouse podocytes | *In situ* proximity ligation assay | n.d. | Heavy chain of clathrin | Interaction rate increased by 40% under insulin treatment relative to starved state | [14] |
| E3 ubiquitin-protein ligase CBL | | TA adipocyte library | Y2H screening | n.d. | Proline‐rich region of CBL | Regulates endosome morphology and PDGF‐Rβ degradation (induced by PDGF) | [12] |
|  | | ° Jurkat total cell lysates  ° Recombinant protein | GST pull-down assay | Peptide array far-western blots | SH3-1 and SH3-2, but not SH3-3 domain of CD2AP | Role in EGF receptor endocytosis | [84] |
| Endophilin | | MDA-MB-231 cell lysates | Co-IP – WB | n.d. | CD2AP proline-rich sequence 1 | Constitutive interaction, unaffected by EGF | [11] |
| Golgi associated, gamma adaptin ear containing ARF binding protein 2 (GGA2) | | Mouse podocytes | Co-IP – WB | Duolink *in situ* proximity ligation assay | n.d. | ° Interaction rate increases by 35% after insulin treatment  ° CDAP inversely impacts GGA2–clathrin binding -> role in trafficking or uncoating | [14] |
| Intercellular adhesion molecule-1 (ICAM-1) | | ° TNF-α-stimulated primary HUVECs  ° HeLa cells | Pull-out clustering experiment | Pull down – WB, co-localization | Intracellular region of ICAM-1 | ° Inhibits ICAM-1 clustering -> transcellular transmigration and inflammation  ° Rac1 and mechanical force negatively control interaction | [85] |
| Nephrin | | HEK 293T cells | Co-IP – WB | n.d. | n.d. | Interaction increases a PI3K dependent activation of Akt | [86] |
| p85 (regulatory subunit of PI3K) | | HEK 293T cells | Co-IP – WB | n.d. | N-terminal portion (amino acids 1 to 334) of CD2AP | ° Activation of Akt (P-Ser-473)  ° Anti-apoptotic (Bad-P: Ser-112/136) | [86] |
| PAWS1 | | ° HEK293 cells  ° U2OS cells | Co-IP – MS | Co-localization | PAWS1 DUF1669 domain (amino acids 151–291) | n.d. | [87] |
| Programmed cell death 6-interacting protein (ALIX) | | Recombinant proteins | Peptide array | Permutation array (20 AA, GST-based) | CD2AP SH3-3 domain | ALIX is a component of the ESCRT, and is, together with SDC1/4 and SDCBP, involved in exosome biogenesis | [84] |
| Rab4 (active) | | ° TA adipocyte library  ° 293 cells | Y2H screening | GST pull-down assay – WB | C‐terminal end (AA 539 and 637) of CD2AP | Role in endosome morphology and transport to degradative pathway | [12] |
| Ras and Rab Interactor 3 (RIN3) | | ° Recombinant protein  ° HEK293 kidney cells  ° Podocytes | Peptide array | Co-IP and GST pull-down – WB, crystallography | CD2AP’s SH3-1 and SH3-2 domain interact with RIN3’s Px(P/A)xPR motif | Interaction entails localization at Rab5a^+^ early endosomes | [84] |
| Ras-related C3 botulinum toxin substrate 1 (Rac1) | | HeLa cells, Jurkat T-cells, primary HUVECs and CD34^+^ cells | Co-IP – MS | GST pull-down assay, co-localization | C-terminal Pro-rich/poly-basic Rac1 domain x CD2AP N-terminal, SH3 domain | CD2AP links Rac1 to CAPZ and cortactin | [88] |
| SH-2 containing inositol 5' poly-phosphatase 1 (SHIP1) | | human plasmacytoid dendritic cells | Co-IP – MS | HA pull-down assay – WB | CD2AP’s first SH3 domain binds SHIP1 P-rich domain | Ubiquitination and degradation of immunoreceptors -> microglial endocytosis | [89] |
| Tropomyosin receptor kinase A (TrkA) | | NGF-stimulated PC12 cells | Co-localization | Co-IP – WB | n.d. | Impacts downstream PI3K/AKT but not MAPK/ERK activity; interacts with p85 to recruit it to TrkA (endocytosis) | [15] |
| Vascular cell adhesion protein 1 (VCAM-1) | | TNF-α-stimulated primary HUVECs | Pull down – WB | n.d. | Intracellular region of VCAM-1 | Less strong interaction as between CD2AP and ICAM-1 | [85] |
| **(PI)CALM interactors** | |  |  |  |  |  |  |
| ABC transporter P-glycoprotein (ABCB1/P-gp) | | ° Brain endothelial cell line bEnd.3  ° Mouse capillaries | Co-IP – WB | Co–localization | n.d. | Functional interplay with LRP1and the rapid transcytosis of Aβ | [90] |
| AP-1 complex-associated regulatory protein (AP1AR) | | LPS-stimulated bone marrow-derived mouse dendritic cells | Co-IP – MS | n.d. | n.d. | Regulation of MYD88-dependent inflammatory signaling | [91] |
| Clathrin heavy chain | | HeLa cells | Co-IP – WB | n.d. | C-terminal epiptope PICALM + conformational epitope | Clathrin-mediated receptor trafficking TGN – endosomes: transferrin & EGF receptors | [20] |
|  | | Recombinant proteins | Peptide array overlay assay | Mutation analysis | PICALM highest affinity sequence LDSSLANLVGNLGIG | n.d. | [92] |
| CALM-interactor expressed in thymus and spleen (CATS) | | ° HeLa cDNA  ° Recombinant proteins  ° HEK293T cells | Y2H screening | GST pull-down assay – WB, Co-IP – WB | Amino acids 221–294 of CATS and amino acids 294–335 of CALM involved | Cats predominantly expressed in lymphoid organs thymus, spleen and colon, but also in brain although to a lesser extend | [93] |
| Complex adaptor protein 2 (AP2) | | ° HeLa cells  ° APP_swe_/PS1_ΔE9_ brains | Co-IP – MS | Anti-GFP Co-IP – MS | n.d. | AP-2/PICALM complex cross-links LC3 to APP-CTF, enabling autophagic degradation | [94] |
|  | | HeLa and N2a cell lysates | GST pull-down assay – WB | n.d. | PICALM ANTH domain and C-terminal region | n.d. | [95] |
|  | | HeLa cells | GST pull-down assay – WB | n.d. | AP2 α‐appendage domain: 420DPF, 375DIF & 489FESVF | n.d. | [18] |
| Four and a half LIM domain protein 2 | | ° Recombinant proteins  ° HEK293T cells | GST pull-down assay – WB | Co-IP – WB, colocalization experiments | Amino acids 294–335 of CALM involved | Binding reduces FHL2 transcriptional activation capacity by half (FOXO1 is one downstream inhibited protein of FHL2) | [96] |
| Low density lipoprotein receptor related protein-1 (LRP1) | | Primary frontal cortex human brain endothelial cells | Co-IP – WB | Proximity ligation assay | PICALM interacts with the YXXL motif of the LRP1 C-terminus | PICALM knockdown: basolateral–to–apical trans–endothelial transport of Aβ_40_ (Aβ–LRP1 endocytosis) abrogated by 85% | [22] |
|  |  | ° Brain endothelial cell line bEnd.3  ° Mouse capillaries | Co-IP – WB | Co–localization | n.d. | Clathrin–linked endocytosis of Aβ–LRP1- ABCB1/P-gp complex to early and sorting endosomes for transcytosis | [90] |
| Nicastrin of the γ-secretase complex | | HeLa and N2a cell lysates | GST pull-down assay – WB | n.d. | PICALM ANTH domain | ° Nicastrin cell surface levels influenced by PICALM-induced endocytosis-degradation  ° VAMP8 competition for epitope binding | [95] |
| Paired helical filament P-tau | | LOAD brain lysates | Co-IP – WB | n.d. | n.d. | Remark: PICALM is not an integral element of paired helical filaments or Aβ plaques | [30] |
| Phosphatidylinositol-4,5- bisphosphate | | Recombinant protein | X-ray crystallography | Sedimentation assays | NH2-terminal domain of PICALM (lysine-rich motif) | n.d. | [97] |
| Rab5 | | Frontal cortex human brain endothelial cells | Co-IP – WB | Co–localization | n.d. | PICALM knockdown reduces Rab5 GTPase’s activity by 80% | [22] |
| Rab11 | | Frontal cortex human brain endothelial cells | Co-IP – WB | Co–localization | n.d. | PICALM knockdown reduces Rab11 GTPase’s activity by 95% | [22] |
| Vesicle-associated membrane protein 2 (VAMP2) | | HeLa cells | Co-IP – WB | n.d. | PICALM ANTH domain (K_D_ of ∼43 μM) | PICALM knockdown stops endocytosis of HA-VAMP2 | [98] |
| VAMP3 | | HeLa cells | Co-IP – WB | n.d. | PICALM ANTH domain (K_D_ of ∼46 μM) | PICALM knockdown stops endocytosis of HA-VAMP3 | [98] |
| VAMP 8 | | HeLa cells | Co-IP – WB | n.d. | Helix α11 in PICALM (1-289) binds to residues 10-41 of VAMP8 (K_D_ of ∼18 μM) | ° PICALM^-/-^ stops HA-VAMP8 endocytosis  ° VAMP8 and PtdIns(4,5)P2 bind together -> order of magnitude decrease in K_D_ | [98] |
|  | | HeLa and N2a cell lysates | GST pull-down assay – WB | n.d. | PICALM ANTH domain | Competes with nicastrin for epitope binding | [95] |
| **PLD3 interactors** | |  |  |  |  |  |  |
| APP | | HEK293T | Co-IP – WB | n.d. | n.d. | PLD3 influences APP processing | [42] |
| Cathepsin B/L | | ° Mouse brain lysates  ° Mouse embryonic fibroblasts | Functional analyses | Functional analyses | n.d. | Enables fast degradation of the N-terminal membrane-bound PLD3 part; no effect on the glycosylated luminal polypeptide | [35] |
| Progranulin | | ° HEK293, SK-N-SH cells  ° AD brain tissue | Co-IP – WB | Co-expression, co-localization | n.d. | Possible shared endosomal transport role | [43] |

**References**

1. Leprince C, Le Scolan E, Meunier B, Fraisier V, Brandon N, De Gunzburg J, et al. Sorting nexin 4 and amphiphysin 2, a new partnership between endocytosis and intracellular trafficking. J Cell Sci. 2003;116 Pt 10:1937–48.

2. Nakajo A, Yoshimura S, Togawa H, Kunii M, Iwano T, Izumi A, et al. EHBP1L1 coordinates Rab8 and Bin1 to regulate apical-directed transport in polarized epithelial cells. J Cell Biol. 2016;212:297–306.

3. Calafate S, Flavin W, Verstreken P, Moechars D. Loss of Bin1 Promotes the Propagation of Tau Pathology. Cell Rep. 2016;17:931–40.

4. Butler MH, David C, Ochoa GC, Freyberg Z, Daniell L, Grabs D, et al. Amphiphysin II (SH3P9; BIN1), a member of the amphiphysin/Rvs family, is concentrated in the cortical cytomatrix of axon initial segments and nodes of ranvier in brain and around T tubules in skeletal muscle. J Cell Biol. 1997;137:1355–67.

5. DuHadaway JB, Lynch FJ, Brisbay S, Bueso-Ramos C, Troncoso P, McDonnell T, et al. Immunohistochemical analysis of Bin1/Amphiphysin II in human tissues: diverse sites of nuclear expression and losses in prostate cancer. J Cell Biochem. 2003;88:635–42.

6. Adams SL, Tilton K, Kozubek JA, Seshadri S, Delalle I. Subcellular Changes in Bridging Integrator 1 Protein Expression in the Cerebral Cortex During the Progression of Alzheimer Disease Pathology. J Neuropathol Exp Neurol. 2016;75:779–90.

7. Karch CM, Jeng AT, Nowotny P, Cady J, Cruchaga C, Goate AM. Expression of Novel Alzheimer’s Disease Risk Genes in Control and Alzheimer’s Disease Brains. PLoS One. 2012;7:e50976.

8. De Rossi P, Buggia-Prévot V, Clayton BLL, Vasquez JB, van Sanford C, Andrew RJ, et al. Predominant expression of Alzheimer’s disease-associated BIN1 in mature oligodendrocytes and localization to white matter tracts. Mol Neurodegener. 2016;11:59.

9. Chapuis J, Hansmannel F, Gistelinck M, Mounier A, Van Cauwenberghe C, Kolen K V, et al. Increased expression of BIN1 mediates Alzheimer genetic risk by modulating tau pathology. Mol Psychiatry. 2013;18:1225–34.

10. De Rossi P, Buggia-Prevot V, Andrew R, Krause S, Woo E, Nelson P, et al. BIN1 localization is distinct from Tau tangles in Alzheimer’s disease. Matters. 2017.

11. Lynch DK, Winata SC, Lyons RJ, Hughes WE, Lehrbach GM, Wasinger V, et al. A Cortactin-CD2-associated protein (CD2AP) complex provides a novel link between epidermal growth factor receptor endocytosis and the actin cytoskeleton. JBC. 2003;278:21805–13.

12. Cormont M, Metón I, Mari M, Monzo P, Keslair F, Gaskin C, et al. CD2AP/CMS regulates endosome morphology and traffic to the degradative pathway through its interaction with Rab4 and c-Cbl. Traffic. 2003;4:97–112.

13. Ubelmann F, Burrinha T, Salavessa L, Gomes R, Ferreira C, Moreno N, et al. Bin1 and CD2AP polarise the endocytic generation of beta-amyloid. EMBO Rep. 2017;18:102–22.

14. Tolvanen TA, Dash SN, Polianskyte-Prause Z, Dumont V, Lehtonen S. Lack of CD2AP disrupts Glut4 trafficking and attenuates glucose uptake in podocytes. J Cell Sci. 2015;128:4588–600.

15. Harrison BJ, Venkat G, Lamb JL, Hutson TH, Drury C, Rau KK, et al. The Adaptor Protein CD2AP Is a Coordinator of Neurotrophin Signaling-Mediated Axon Arbor Plasticity. J Neurosci. 2016;36:4259–75.

16. Lehtonen S, Tienari J, Londesborough A, Pirvola U, Ora A, Reima I, et al. CD2-associated protein is widely expressed and differentially regulated during embryonic development. Differentiation. 2008;76:506–17.

17. Li C, Ruotsalainen V, Tryggvason K, Shaw AS, Miner JH. CD2AP is expressed with nephrin in developing podocytes and is found widely in mature kidney and elsewhere. Am J Physiol Renal Physiol. 2000;279:F785-92.

18. Meyerholz A, Hinrichsen L, Groos S, Esk P-C, Brandes G, Ungewickell EJ. Effect of clathrin assembly lymphoid myeloid leukemia protein depletion on clathrin coat formation. Traffic. 2005;6:1225–34.

19. Moreau K, Fleming A, Imarisio S, Lopez Ramirez A, Mercer JL, Jimenez-Sanchez M, et al. PICALM modulates autophagy activity and tau accumulation. Nat Commun. 2014;5:4998.

20. Tebar F, Bohlander SK, Sorkin A. Clathrin assembly lymphoid myeloid leukemia (CALM) protein: localization in endocytic-coated pits, interactions with clathrin, and the impact of overexpression on clathrin-mediated traffic. Mol Biol Cell. 1999;10:2687–702.

21. Thomas RS, Henson A, Gerrish A, Jones L, Williams J, Kidd EJ. Decreasing the expression of PICALM reduces endocytosis and the activity of β-secretase: implications for Alzheimer’s disease. BMC Neurosci. 2016;17:50.

22. Zhao Z, Sagare AP, Ma Q, Halliday MR, Kong P, Kisler K, et al. Central role for PICALM in amyloid-β blood-brain barrier transcytosis and clearance. Nat Neurosci. 2015;18:978–87.

23. Bushlin I, Petralia RS, Wu F, Harel A, Mughal MR, Mattson MP, et al. Clathrin assembly protein AP180 and CALM differentially control axogenesis and dendrite outgrowth in embryonic hippocampal neurons. J Neurosci. 2008;28:10257–71.

24. Petralia RS, Yao PJ. AP180 and CALM in the developing hippocampus: expression at the nascent synapse and localization to trafficking organelles. J Comp Neurol. 2007;504:314–27.

25. Schwartz CM, Cheng A, Mughal MR, Mattson MP, Yao PJ. Clathrin assembly proteins AP180 and CALM in the embryonic rat brain. J Comp Neurol. 2010;518:3803–18.

26. Yao PJ, Petralia RS, Bushlin I, Wang Y, Furukawa K. Synaptic distribution of the endocytic accessory proteins AP180 and CALM. J Comp Neurol. 2005;481:58–69.

27. Thomas RS, Alsaqati M, Bice JS, Hvoslef-Eide M, Good MA, Kidd EJ. Alterations in endocytic protein expression with increasing age in the transgenic APP695 V717I London mouse model of amyloid pathology: implications for Alzheimer’s disease. Neuroreport. 2017;28:963–8.

28. Xiao Q, Gil S-C, Yan P, Wang Y, Han S, Gonzales E, et al. Role of phosphatidylinositol clathrin assembly lymphoid-myeloid leukemia (PICALM) in intracellular amyloid precursor protein (APP) processing and amyloid plaque pathogenesis. JBC. 2012;287:21279–89.

29. Ando K, Tomimura K, Sazdovitch V, Suain V, Yilmaz Z, Authelet M, et al. Level of PICALM, a key component of clathrin-mediated endocytosis, is correlated with levels of phosphotau and autophagy-related proteins and is associated with tau inclusions in AD, PSP and Pick disease. Neurobiol Dis. 2016;94:32–43.

30. Ando K, Brion J-P, Stygelbout V, Suain V, Authelet M, Dedecker R, et al. Clathrin adaptor CALM/PICALM is associated with neurofibrillary tangles and is cleaved in Alzheimer’s brains. Acta Neuropathol. 2013;125:861–78.

31. Parikh I, Fardo DW, Estus S. Genetics of PICALM expression and Alzheimer’s disease. PLoS One. 2014;9:e91242.

32. Baig S, Joseph SA, Tayler H, Abraham R, Owen MJ, Williams J, et al. Distribution and expression of picalm in Alzheimer disease. J Neuropathol Exp Neurol. 2010;69:1071–7.

33. Munck A, Böhm C, Seibel NM, Hashemol Hosseini Z, Hampe W. Hu-K4 is a ubiquitously expressed type 2 transmembrane protein associated with the endoplasmic reticulum. FEBS J. 2005;272:1718–26.

34. Mukadam AS, Breusegem SY, Seaman MNJ. Analysis of novel endosome-to-Golgi retrieval genes reveals a role for PLD3 in regulating endosomal protein sorting and amyloid precursor protein processing. CMLS. 2018;75:2613–25.

35. Gonzalez AC, Schweizer M, Jagdmann S, Bernreuther C, Reinheckel T, Saftig P, et al. Unconventional Trafficking of Mammalian Phospholipase D3 to Lysosomes. Cell Rep. 2018;22:1040–53.

36. Valli A, Rodriguez M, Moutsianas L, Fischer R, Fedele V, Huang H-L, et al. Hypoxia induces a lipogenic cancer cell phenotype via HIF1α-dependent and -independent pathways. Oncotarget. 2015;6:1920–41.

37. Osisami M, Ali W, Frohman MA. A role for phospholipase D3 in myotube formation. PLoS One. 2012;7:e33341.

38. Fazzari P, Horre K, Arranz AM, Frigerio CS, Saito T, Saido TC, et al. PLD3 gene and processing of APP. Nature. 2017;541:E1–2.

39. Pedersen KM, Finsen B, Celis JE, Jensen NA. Expression of a novel murine phospholipase D homolog coincides with late neuronal development in the forebrain. JBC. 1998;273:31494–504.

40. Kent DG, Copley MR, Benz C, Wöhrer S, Dykstra BJ, Ma E, et al. Prospective isolation and molecular characterization of hematopoietic stem cells with durable self-renewal potential. Blood. 2009;113:6342–50.

41. Nibbeling EAR, Duarri A, Verschuuren-Bemelmans CC, Fokkens MR, Karjalainen JM, Smeets CJLM, et al. Exome sequencing and network analysis identifies shared mechanisms underlying spinocerebellar ataxia. Brain. 2017;140:2860–78.

42. Cruchaga C, Karch CM, Jin SC, Benitez BA, Cai Y, Guerreiro R, et al. Rare coding variants in the phospholipase D3 gene confer risk for Alzheimer’s disease. Nature. 2014;505:550–4.

43. Satoh J-I, Kino Y, Yamamoto Y, Kawana N, Ishida T, Saito Y, et al. PLD3 is accumulated on neuritic plaques in Alzheimer’s disease brains. Alzheimers Res Ther. 2014;6:70.

44. Seshadri S, Fitzpatrick AL, Ikram MA, DeStefano AL, Gudnason V, Boada M, et al. Genome-wide analysis of genetic loci associated with Alzheimer disease. JAMA. 2010;303:1832–40.

45. Dong X, Zhang L, Meng Q, Gao Q. Association Between Interleukin-1A, Interleukin-1B, and Bridging integrator 1 Polymorphisms and Alzheimer’s Disease: a standard and Cumulative Meta-analysis. Mol Neurobiol. 2017;54:736–47.

46. Zhang X, Yu J-T, Li J, Wang C, Tan L, Liu B, et al. Bridging Integrator 1 (BIN1) Genotype Effects on Working Memory, Hippocampal Volume, and Functional Connectivity in Young Healthy Individuals. Neuropsychopharmacology. 2015;40:1794–803.

47. Rodríguez-Rodríguez E, Sánchez-Juan P, Vázquez-Higuera JL, Mateo I, Pozueta A, Berciano J, et al. Genetic risk score predicting accelerated progression from mild cognitive impairment to Alzheimer’s disease. J Neural Transm. 2013;120:807–12.

48. Li J-Q, Wang H-F, Zhu X-C, Sun F-R, Tan M-S, Tan C-C, et al. GWAS-Linked Loci and Neuroimaging Measures in Alzheimer’s Disease. Mol Neurobiol. 2017;54:146–53.

49. Wachinger C, Nho K, Saykin AJ, Reuter M, Rieckmann A, Alzheimer’s Disease Neuroimaging Initiative. A Longitudinal Imaging Genetics Study of Neuroanatomical Asymmetry in Alzheimer’s Disease. Biol Psychiatry. 2018.

50. Greenbaum L, Ravona-Springer R, Lubitz I, Schmeidler J, Cooper I, Sano M, et al. Potential contribution of the Alzheimer’s disease risk locus BIN1 to episodic memory performance in cognitively normal Type 2 diabetes elderly. Eur Neuropsychopharmacol. 2016;26:787–95.

51. Naj AC, Jun G, Beecham GW, Wang L-S, Vardarajan BN, Buros J, et al. Common variants at MS4A4/MS4A6E, CD2AP, CD33 and EPHA1 are associated with late-onset Alzheimer’s disease. Nat Genet. 2011;43:436–41.

52. Wijsman EM, Pankratz ND, Choi Y, Rothstein JH, Faber KM, Cheng R, et al. Genome-wide association of familial late-onset Alzheimer’s disease replicates BIN1 and CLU and nominates CUGBP2 in interaction with APOE. PLoS Genet. 2011;7:e1001308.

53. Biffi A, Anderson CD, Desikan RS, Sabuncu M, Cortellini L, Schmansky N, et al. Genetic variation and neuroimaging measures in Alzheimer disease. Arch Neurol. 2010;67:677–85.

54. Shulman JM, Chen K, Keenan BT, Chibnik LB, Fleisher A, Thiyyagura P, et al. Genetic susceptibility for Alzheimer disease neuritic plaque pathology. JAMA Neurol. 2013;70:1150–7.

55. Hu X, Pickering E, Liu YC, Hall S, Fournier H, Katz E, et al. Meta-analysis for genome-wide association study identifies multiple variants at the BIN1 locus associated with late-onset Alzheimer’s disease. PLoS One. 2011;6:e16616.

56. Hollingworth P, Harold D, Sims R, Gerrish A, Lambert J-C, Carrasquillo MM, et al. Common variants at ABCA7, MS4A6A/MS4A4E, EPHA1, CD33 and CD2AP are associated with Alzheimer’s disease. Nat Genet. 2011;43:429–35.

57. Lazaris A, Hwang KS, Goukasian N, Ramirez LM, Eastman J, Blanken AE, et al. Alzheimer risk genes modulate the relationship between plasma apoE and cortical PiB binding. Neurol Genet. 2015;1:e22.

58. Jun G, Naj AC, Beecham GW, Wang L-S, Buros J, Gallins PJ, et al. Meta-analysis confirms CR1, CLU, and PICALM as alzheimer disease risk loci and reveals interactions with APOE genotypes. Arch Neurol. 2010;67:1473–84.

59. Seripa D, Panza F, Paroni G, D’Onofrio G, Bisceglia P, Gravina C, et al. Role of CLU, PICALM, and TNK1 Genotypes in Aging With and Without Alzheimer’s Disease. Mol Neurobiol. 2018;55:4333–44.

60. Schjeide B-MM, Schnack C, Lambert J-C, Lill CM, Kirchheiner J, Tumani H, et al. The role of clusterin, complement receptor 1, and phosphatidylinositol binding clathrin assembly protein in Alzheimer disease risk and cerebrospinal fluid biomarker levels. Arch Gen Psychiatry. 2011;68:207–13.

61. Harold D, Abraham R, Hollingworth P, Sims R, Gerrish A, Hamshere ML, et al. Genome-wide association study identifies variants at CLU and PICALM associated with Alzheimer’s disease. Nat Genet. 2009;41:1088–93.

62. Santos-Rebouças CB, Gonçalves AP, Dos Santos JM, Abdala BB, Motta LB, Laks J, et al. rs3851179 Polymorphism at 5’ to the PICALM Gene is Associated with Alzheimer and Parkinson Diseases in Brazilian Population. Neuromolecular Med. 2017;19:293–9.

63. Mengel-From J, Christensen K, McGue M, Christiansen L. Genetic variations in the CLU and PICALM genes are associated with cognitive function in the oldest old. Neurobiol Aging. 2011;32:554.e7-11.

64. Ferencz B, Laukka EJ, Welmer A-K, Kalpouzos G, Angleman S, Keller L, et al. The benefits of staying active in old age: physical activity counteracts the negative influence of PICALM, BIN1, and CLU risk alleles on episodic memory functioning. Psychol Aging. 2014;29:440–9.

65. Carrasquillo MM, Crook JE, Pedraza O, Thomas CS, Pankratz VS, Allen M, et al. Late-onset Alzheimer’s risk variants in memory decline, incident mild cognitive impairment, and Alzheimer’s disease. Neurobiol Aging. 2015;36:60–7.

66. Morgen K, Ramirez A, Frölich L, Tost H, Plichta MM, Kölsch H, et al. Genetic interaction of PICALM and APOE is associated with brain atrophy and cognitive impairment in Alzheimer’s disease. Alzheimers Dement. 2014;10 5 Suppl:S269-76.

67. Furney SJ, Simmons A, Breen G, Pedroso I, Lunnon K, Proitsi P, et al. Genome-wide association with MRI atrophy measures as a quantitative trait locus for Alzheimer’s disease. Mol Psychiatry. 2011;16:1130–8.

68. Lee JH, Cheng R, Barral S, Reitz C, Medrano M, Lantigua R, et al. Identification of novel loci for Alzheimer disease and replication of CLU, PICALM, and BIN1 in Caribbean Hispanic individuals. Arch Neurol. 2011;68:320–8.

69. Engelman CD, Darst BF, Bilgel M, Vasiljevic E, Koscik RL, Jedynak BM, et al. The effect of rare variants in TREM2 and PLD3 on longitudinal cognitive function in the Wisconsin Registry for Alzheimer’s Prevention. Neurobiol Aging. 2018;66:177.e1-177.e5.

70. van der Lee SJ, Holstege H, Wong TH, Jakobsdottir J, Bis JC, Chouraki V, et al. PLD3 variants in population studies. Nature. 2015;520:E2–3.

71. Dräger NM, Nachman E, Winterhoff M, Brühmann S, Shah P, Katsinelos T, et al. Bin1 directly remodels actin dynamics through its BAR domain. EMBO Rep. 2017;18:2051–66.

72. Miyagawa T, Ebinuma I, Morohashi Y, Hori Y, Young Chang M, Hattori H, et al. BIN1 regulates BACE1 intracellular trafficking and amyloid-β production. Hum Mol Genet. 2016;25:2948–58.

73. Berggård T, Arrigoni G, Olsson O, Fex M, Linse S, James P. 140 mouse brain proteins identified by Ca2+-calmodulin affinity chromatography and tandem mass spectrometry. J Proteome Res. 2006;5:669–87.

74. Xu B, Fu Y, Liu Y, Agvanian S, Wirka RC, Baum R, et al. The ESCRT-III pathway facilitates cardiomyocyte release of cBIN1-containing microparticles. PLoS Biol. 2017;15:e2002354.

75. Ramjaun AR, Micheva KD, Bouchelet I, McPherson PS. Identification and characterization of a nerve terminal-enriched amphiphysin isoform. JBC. 1997;272:16700–6.

76. Meunier B, Quaranta M, Daviet L, Hatzoglou A, Leprince C. The membrane-tubulating potential of amphiphysin 2/BIN1 is dependent on the microtubule-binding cytoplasmic linker protein 170 (CLIP-170). Eur J Cell Biol. 2009;88:91–102.

77. Takeda T, Kozai T, Yang H, Ishikuro D, Seyama K, Kumagai Y, et al. Dynamic clustering of dynamin-amphiphysin helices regulates membrane constriction and fission coupled with GTP hydrolysis. Elife. 2018;7.

78. Desrochers G, Cappadocia L, Lussier-Price M, Ton A-T, Ayoubi R, Serohijos A, et al. Molecular basis of interactions between SH3 domain-containing proteins and the proline-rich region of the ubiquitin ligase Itch. JBC. 2017;292:6325–38.

79. Sakamuro D, Elliott KJ, Wechsler-Reya R, Prendergast GC. BIN1 is a novel MYC-interacting protein with features of a tumour suppressor. Nat Genet. 1996;14:69–77.

80. Kajiho H, Saito K, Tsujita K, Kontani K, Araki Y, Kurosu H, et al. RIN3: a novel Rab5 GEF interacting with amphiphysin II involved in the early endocytic pathway. J Cell Sci. 2003;116 Pt 20:4159–68.

81. Malki I, Cantrelle F-X, Sottejeau Y, Lippens G, Lambert J-C, Landrieu I. Regulation of the interaction between the neuronal BIN1 isoform 1 and Tau proteins - role of the SH3 domain. FEBS J. 2017;284:3218–29.

82. Monzo P, Gauthier NC, Keslair F, Loubat A, Field CM, Le Marchand-Brustel Y, et al. Clues to CD2-associated protein involvement in cytokinesis. Mol Biol Cell. 2005;16:2891–902.

83. Dustin ML, Olszowy MW, Holdorf AD, Li J, Bromley S, Desai N, et al. A Novel Adaptor Protein Orchestrates Receptor Patterning and Cytoskeletal Polarity in T-Cell Contacts. Cell. 1998;94:667–77.

84. Rouka E, Simister PC, Janning M, Kumbrink J, Konstantinou T, Muniz JRC, et al. Differential Recognition Preferences of the Three Src Homology 3 (SH3) Domains from the Adaptor CD2-associated Protein (CD2AP) and Direct Association with Ras and Rab Interactor 3 (RIN3). JBC. 2015;290:25275–92.

85. Schaefer A, van Duijn TJ, Majolee J, Burridge K, Hordijk PL. Endothelial CD2AP Binds the Receptor ICAM-1 To Control Mechanosignaling, Leukocyte Adhesion, and the Route of Leukocyte Diapedesis In Vitro. J Immunol. 2017;198:4823–36.

86. Huber TB, Hartleben B, Kim J, Schmidts M, Schermer B, Keil A, et al. Nephrin and CD2AP associate with phosphoinositide 3-OH kinase and stimulate AKT-dependent signaling. Mol Cell Biol. 2003;23:4917–28.

87. Cummins TD, Wu KZL, Bozatzi P, Dingwell KS, Macartney TJ, Wood NT, et al. PAWS1 controls cytoskeletal dynamics and cell migration through association with the SH3 adaptor CD2AP. J Cell Sci. 2018;131:jcs202390.

88. van Duijn TJ, Anthony EC, Hensbergen PJ, Deelder AM, Hordijk PL. Rac1 recruits the adapter protein CMS/CD2AP to cell-cell contacts. JBC. 2010;285:20137–46.

89. Bao M, Hanabuchi S, Facchinetti V, Du Q, Bover L, Plumas J, et al. CD2AP/SHIP1 complex positively regulates plasmacytoid dendritic cell receptor signaling by inhibiting the E3 ubiquitin ligase Cbl. J Immunol. 2012;189:786–92.

90. Storck SE, Hartz AMS, Bernard J, Wolf A, Kachlmeier A, Mahringer A, et al. The concerted amyloid-beta clearance of LRP1 and ABCB1/P-gp across the blood-brain barrier is linked by PICALM. Brain Behav Immun. 2018.

91. Mertins P, Przybylski D, Yosef N, Qiao J, Clauser K, Raychowdhury R, et al. An Integrative Framework Reveals Signaling-to-Transcription Events in Toll-like Receptor Signaling. Cell Rep. 2017;19:2853–66.

92. Moshkanbaryans L, Xue J, Wark JR, Robinson PJ, Graham ME. A Novel Sequence in AP180 and CALM Promotes Efficient Clathrin Binding and Assembly. PLoS One. 2016;11:e0162050.

93. Archangelo LF, Gläsner J, Krause A, Bohlander SK. The novel CALM interactor CATS influences the subcellular localization of the leukemogenic fusion protein CALM/AF10. Oncogene 2006 2529. 2006;25:4099.

94. Tian Y, Chang JC, Fan EY, Flajolet M, Greengard P. Adaptor complex AP2/PICALM, through interaction with LC3, targets Alzheimer’s APP-CTF for terminal degradation via autophagy. PNAS U S A. 2013;110:17071–6.

95. Kanatsu K, Morohashi Y, Suzuki M, Kuroda H, Watanabe T, Tomita T, et al. Decreased CALM expression reduces Aβ42 to total Aβ ratio through clathrin-mediated endocytosis of γ-secretase. Nat Commun. 2014;5:3386.

96. Pašaliç Z, Greif PA, Jurinoviç V, Mulaw M, Kakadia PM, Tizazu B, et al. FHL2 interacts with CALM and is highly expressed in acute erythroid leukemia. Blood Cancer J. 2011;1:e42.

97. Ford MG, Pearse BM, Higgins MK, Vallis Y, Owen DJ, Gibson A, et al. Simultaneous binding of PtdIns(4,5)P2 and clathrin by AP180 in the nucleation of clathrin lattices on membranes. Science. 2001;291:1051–5.

98. Miller SE, Sahlender DA, Graham SC, Höning S, Robinson MS, Peden AA, et al. The molecular basis for the endocytosis of small R-SNAREs by the clathrin adaptor CALM. Cell. 2011;147:1118–31.
