# Supplementary figures and images for: Endo-lysosomal dysregulations and late-onset Alzheimer’s disease: impact of genetic risk factors
Source: Mol Neurodegener. 2019 Jun 3;14:20. doi: 10.1186/s13024-019-0323-7 (PMC6547588; doi:10.1186/s13024-019-0323-7)

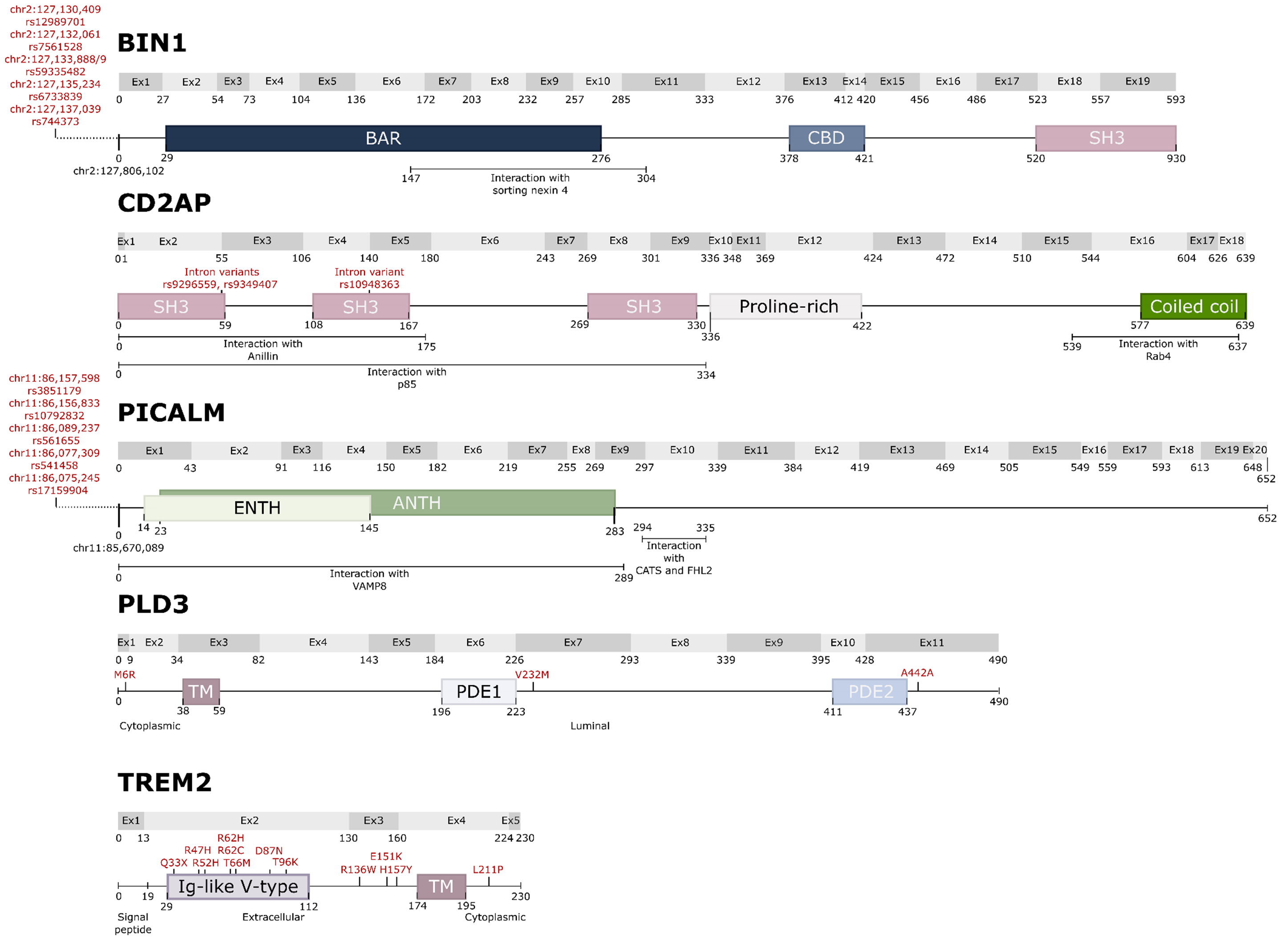

Supplement: Supplementary file 2 — Figure S1. Schematic representation of BIN1, CD2AP, PICALM, PLD3 and TREM2 structures and the relative positions of known SNPs. Abbreviations: ANTH, AP180 N-terminal homology; BAR, bin-amphiphysin-rvs; CATS, family with sequence similarity 64 member A; CBD, clathrin-binding domain; ENTH, epsin N-terminal homology; FHL2, four and a half LIM domains 2; PDE1, phosphodiesterase type 1; SH3, SRC homology 3; VAMP8, vesicle-associated membrane protein 8. (TIF 1027 kb) [file 13024_2019_323_MOESM2_ESM.tif]
